# Adjuvant treatment with the bacterial lysate (OM-85) improves management of atopic dermatitis: A randomized study

**DOI:** 10.1371/journal.pone.0161555

**Published:** 2017-03-23

**Authors:** Christine Bodemer, Gerard Guillet, Frederic Cambazard, Franck Boralevi, Stefania Ballarini, Christian Milliet, Paola Bertuccio, Carlo La Vecchia, Jean-François Bach, Yves de Prost

**Affiliations:** 1 Assistance Publique-Hôpitaux de Paris, Hôpital Necker-Enfants Malades, Department of Dermatology, Paris, France; 2 Université de Poitiers, Department of Dermatology, Poitiers, France; 3 Université de St-Etienne, Hôpital de St-Etienne, Department of Dermatology, Saint-Etienne, France; 4 Université de Bordeaux, Hôpital Pellegrin-enfants, Pediatric Dermatology Unit, Bordeaux, France; 5 Vifor Pharma/OM Pharma, Meyrin, Switzerland; 6 Università degli Studi di Milano, Department of Clinical Sciences and Community Health, Milan, Italy; 7 Université Paris Descartes, Sorbone Paris Cité, Paris, France; 8 INSERM U1151- CNRS UMR 8253, INEM (Institut Necker Enfants Malades), Paris, France; Ospedale Maggiore Policlinico, ITALY

## Abstract

**Background:**

Environmental factors play a major role on atopic dermatitis (AD) which shows a constant rise in prevalence in western countries over the last decades. The Hygiene Hypothesis suggesting an inverse relationship between incidence of infections and the increase in atopic diseases in these countries, is one of the working hypothesis proposed to explain this trend.

**Objective:**

This study tested the efficacy and safety of oral administration of the bacterial lysate OM-85 (Broncho-Vaxom^®^, Broncho-Munal^®^, Ommunal^®^, Paxoral^®^, Vaxoral^®^), in the treatment of established AD in children.

**Methods:**

Children aged 6 months to 7 years, with confirmed AD diagnosis, were randomized in a double-blind, placebo-controlled trial to receive, in addition to conventional treatment with emollients and topical corticosteroids, 3.5mg of the bacterial extract OM-85 or placebo daily for 9 months. The primary end-point was the difference between groups in the occurrence of new flares (NF) during the study period, evaluated by Hazard Ratio (HR) derived from conditional Cox proportional hazard regression models accounting for repeated events.

**Results:**

Among the 179 randomized children, 170 were analysed, 88 in the OM-85 and 82 in the placebo group. As expected most children in both treatment groups experienced at least 1 NF during the study period (75 (85%) patients in the OM-85 group and 72 (88%) in the placebo group). Patients treated with OM-85 as adjuvant therapy had significantly fewer and delayed NFs (HR of repeated flares = 0.80; 95% confidence interval (CI): 0.67–0.96), also when potential confounding factors, as family history of atopy and corticosteroids use, were taken into account (HR = 0.82; 95% CI: 0.69–0.98). No major side effect was reported, with comparable and good tolerability for OM-85 and placebo.

**Conclusions:**

Results show an adjuvant therapeutic effect of a well standardized bacterial lysate OM-85 on established AD.

## Introduction

Current treatment of atopic dermatitis (AD) mainly relies on the local application of topical agents, particularly corticosteroids [1]. More rarely, in severe cases, immunosuppressants such as cyclosporin are used [2].

The Hygiene Hypothesis developed in recent years as one of the possibilities [3–5] to explain the predisposing role of improved hygiene in the increase of atopic diseases in industrialized countries has opened new therapeutic perspectives. A causal effect is suggested between the decrease in the incidence of infections and the increase in the incidence of AD and, more generally, of allergic diseases [5, 6]. It was tempting, therefore, to use treatments based on the stimulation of the immune system by derivatives mimicking the effect of bacteria, viruses or parasites as substitute for the “protective” role of infections.

In a first step, it was confirmed that one could effectively prevent experimental models of allergy, such as the development of allergic asthma induced following immunization with ovalbumin, by the administration of killed infectious agents such as mycobacteria [7–9], or bacterial extracts [10–13] or probiotics [14]. This strategy was successfully extended to other models of allergy such as rhinitis [15], food allergy [16] and, interestingly enough, also to experimental autoimmune diseases, such as insulin-dependent diabetes mellitus [17], to which the hygiene hypothesis also applies [5]. Importantly, in experimental models of both allergy and autoimmunity, it has been shown that signaling through single different Toll like receptors (TLR), present at the surface of different immune cells and in particular of antigen-presenting cells (i.e., dendritic cells), fully recapitulates the protective effect of infectious agents [14]. This suggests that it is not the nature of the microbial agent(s) or extract(s) that matters, but rather the type of TLR receptor(s) that are triggered, which by definition may be common between these agents.

Based on these experimental results, attempts have been made to apply this strategy to human AD. For obvious reasons it was decided not to use living infectious agents, even though this was attempted in certain autoimmune diseases, such as multiple sclerosis using a parasite, *Trichuris suis* [18, 19]. In AD it was mainly the use of probiotics that prevailed. Numerous clinical trials have been published with this approach after the pioneering work of the Finnish group of Kalliomaki [20, 21]. The results were the subject of controversy because the data were inconsistently reproduced by different groups [22]. However, our recent meta-analysis concluded that probiotics had a real effect on disease prevention, observed in about 30% of patients [23]. Importantly, these results were mainly obtained when probiotics were administered preventively in children at risk of developing the disease due to atopic heredity. Inconsistent data are presently available on the treatment with probiotics in established AD [24–27]. The probiotic approach is interesting but questionable because of the diversity and lack of standardization of the preparations used.

Another approach, selected in several studies, was to use killed bacteria or bacterial extracts with, however, contrasting results [28–31].

Here we report a new clinically innovative approach using a bacterial lysate (i.e, OM-85). The choice of this agent for our trial was based first, on promising results obtained in experimental dysimmune diseases, showing that its adequate administration could recapitulate the protective effect of infections, according to the Hygiene Hypothesis [17]. Secondly, OM-85 has been extensively used in the clinic as co-medication in the treatment of acute airways infections with an extremely good safety profile, both in adults and very young children in different countries [32, 33].

As described in detail below, our trial was conducted between 2003 and 2006. We are reporting the data today because over the last few years the strategy linked to the use of bacterial derivatives in allergy has regained interest, in particular because of mechanistic studies showing that the bacterial lysate in question is endowed with protective effects linked to stimulation of TLRs [14] (and our unpublished data). This pharmacological effect is novel and timely in the context of the immune mechanisms underlying the Hygiene Hypothesis.

## Methods

The study started in June 2003 and ended in June 2006. This trial was not registered at ClinicalTrials.gov, since only in 2005 the International Committee of Medical Journal Editors (ICMJE) began to require trial registration before the time of first patient enrolment. The clinical trial has been registered in Clinical Trials.gov: https://clinicaltrials.gov/ct2/show/NCT03047954 Registry entry: NCT03047954. The corresponding Clinical Trial Checklist is reported as Supporting information (see S1 Checklist). For transparency, the study protocol was reported as Supporting information (see S1 Text). The study was approved by the French Central Ethics Committee (CCPPRB) of Paris-Necker the March 3, 2003. All parents/legal guardians gave written informed consent prior to enrolment in the study. The study was reported according to CONSORT recommendations [34, 35].

### Study patients

Patients eligible to recruitment were outpatients children of both sexes, aged 6 months to 7 years, with confirmed diagnosis of mild to severe AD, defined by using the UK Working Party’s Diagnostic Criteria for AD [36] and a severity defined by a SCORAD (for SCORing Atopic Dermatitis) [37, 38] between 25 and 70 and an affected body surface area between 15% and 70%. AD patients under systemic steroid and/or immunosuppressive or immune-stimulating therapy within 1 month of study start, or having any immunodeficiency, autoimmune or malignant disease, or those with a known allergy, previous intolerance, known hypersensitivity to the trial drug or to corticoids were ineligible. Participants in another clinical trial and/or treatment with an experimental drug within 3 months of study start were also ineligible.

### Study design and randomization

The study was a prospective, randomized (1:1), double blind, placebo controlled trial of parallel groups, conducted in 8 French hospital centres (that recruited over 70% of the patients) plus 14 French private pediatrician practitioner centres. The number of patients were randomly allocated to treatment groups, according to the random permuted block scheme. In order to balance both treatment arms in relation to the allergic status of the children, SCORAD at entry was taken into account (Block A: SCORAD < 40, Block B: SCORAD ≥ 40). The generation of the random code list including the production of sealed envelopes was performed in a validated environment at the clinical pharmacy department of the sponsor company.

We assessed participants at 6 consecutive visits: enrolment visit (day 0), 4 follow-up visits at 1, 3, 6, 9 months of treatment, and 1 post-treatment visit 12 months after inclusion.

### Treatment

The investigational drug supplied was OM-85, a lyophilized lysate of 21 strains from 8 common respiratory pathogenic microorganisms (i.e. *Haemophilus influenzae*, *Streptococcus pneumoniae*, *Klebsiella ozaenae and pneumoniae*, *Staphylococcus aureus*, *Streptococcus viridans and pyrogenes*, *Neisseria catarrhalis*). Each capsule contained 3.5 mg of lyophilised bacterial extract as active principle. Matched placebo capsules were used as comparative treatment. The dosage was one capsule per day. In young children who could not swallow the whole capsule, it was recommended to open it and pour its content into some liquid (tea, milk, juice, etc.). All children received conventional treatment with emollient and topical corticosteroids. In order to harmonize treatments, the following drugs were prescribed to all children:

- Dexeryl^®^ (emollient): one application daily during the whole study;
- Flixovate^®^ cream (topical corticosteroid): one application daily during 8 days followed by 1 application every 2 days during 8 days and then 1 application every 3 days during 8 days. Flixovate^®^ cream was prescribed at each visit. In case of persistence of the symptoms, the patient was withdrawn from the study. The duration of the study treatment per patient was 9 months, followed by a 3 month follow-up period without treatment. The rationale for the choice of the treatment regimen was to mimic at best, according to the Hygiene Hypothesis, the situation of a microbial environment delivering a chronic, relatively long term, yet safe, stimulus to the immune system to “protect” from disease progression.

### Assessments

#### Primary efficacy parameter

The primary efficacy parameter was the difference between groups (OM-85 *versus* placebo) in the occurrence of AD new flares (NF) during the treatment period. An AD flare was defined as: recurrence of AD lesions rated ≥3 according to global evaluation criteria with a minimum of 15 days from the last flare (Table 1). NFs could be recorded in regular or intermediate visits and between visits (reported by parents to the physician during the subsequent visit).

**Table 1:** New flare definition.

New Flare	Score	Definition
No	0 = remission	No signs of cutaneous inflammatory lesions
No	1 = close to remission	Very slight erythema, and/or infiltration just perceptible.
No	2 = minor lesions	Erythema visible but small and/or slight infiltration/papules
Yes	3 = mild lesions	Erythema of mild intensity, infiltration/papules of mild intensity, pruritus, crusted lesions.
Yes	4 = severe lesions	Marked erythema and/or infiltration/papules, pruritus, crusted lesions.
Yes	5 = higly severe lesions	Intense erythema, infiltration/papule marked with oozing/excoriation.

A Review Committee, in blind conditions (members did not known neither the center nor the real patient’s number), evaluated the pertinence of the primary efficacy variable reported by different investigators in order to reduce at the minimal level:

- non-documented NFs;
- “relapses” (if distance from preceding NF was <15 days);
- “persistent flare” (if repeated);
- false positives, defined as: NF = Intensity ≥3 but SCORAD <25, sum of symptoms <4, pruritus <3;
- false negatives, defined as NF = Intensity <3 but SCORAD >25, sum of symptoms ≥4, pruritus >3, with the help of the patient’s diary (whenever present) or by changing non documented NFs, relapses, persisting with dates very close to visit dates into documented NFs.

The Review Committee members were the study investigator coordinators (2), clinical trial managers (2) and a center representative member (1).

#### Secondary efficacy parameters

Secondary efficacy parameters were the differences between groups in (1) changes in SCORAD over the study period and (2) in the total consumption of corticosteroid.

#### Treatment compliance

Treatment compliance was checked by means of Drug Accountability. The parents/legal guardians were asked to return all unused study medications, including empty boxes blisters at each visit. The returned unused capsules were recorded in the case report form (CRF) by the investigator. The compliance was rated as good if more than 80% of the capsules had been taken, as moderate if equal to 70% to 80%, and as poor, if less than 70% of the capsules had been taken.

#### Tolerability

Overall tolerability of the treatment was assessed at each visit by a general physical status at the time of examination. The presence of any adverse event was registered at each visit, including the potential relationship with the study drug.

### Statistical analysis

#### Analysed population

An intent-to-treat analysis (ITT) was performed, i.e., all randomized subjects, who had taken at least one dose of the trial medication, regardless of their compliance with the protocol, were included in the analysis.

#### Primary efficacy parameter

The efficacy of OM-85 *versus* placebo was tested by means of the Hazard Ratio (HR) estimates, obtained applying conditional Cox proportional hazard regression models accounting for multiple events (i.e., NFs). Among these models, we used the counting process model of Andersen and Gill [39]. The time to a NF was used as the endpoint for the multiple-failure model, with multiple observations per subject depending on the number of events (flares) each subject experienced during the study period. In order to account for within-subject correlation between events, the robust variance ‘sandwich’ estimator was applied [40–42]. The HRs associated with the effect of treatment on repeated occurrence of flares were showed together with their 95% confidence intervals (95% CI). HRs lower than 1 indicate a better result in the OM-85 group compared to placebo (i.e. longer time between flares and less subjects with a high number of flares in the OM-85 group).

A multivariable model was also fit including sex, age (less or more than 3 years), family history of atopy in first degree relatives, and total corticosteroids use as potential confounders of the treatment effect.

A per-protocol analysis was also performed including children from whom data were recovered for the four follow-up visits planned in the protocol and considering only NFs diagnosed during these visits (thus excluding NFs diagnosed during intermediate visits and those reported by parents, for which potential distortion may have had a role despite randomization).

Two supplementary analyses, not initially planned in the study protocol, were performed only for descriptive purpose. The first one compared the number of NFs in each group cumulated from the end of the treatment to visit 6 (i.e. 3 months after the end of treatment) through the Mann-Whitney test. A second not planned analysis was conducted to identify potential differences in the treatment effect across levels of major covariates (i.e. sex, age, family history of atopy in parents and SCORAD at entry). Differences between HR estimates in strata of a covariate were assessed using the Q statistic [43].

#### Secondary efficacy parameters

Differences between groups in the SCORAD evolution over time (from the first to the fourth follow-up visit) were assessed applying a non-parametric analysis of variance (ANOVA) for repeated measures, adjusting for age and sex. A non-parametric ANOVA adjusted for the same potential confounding factors was used to compare total consumption of topical corticosteroids (Flixovate) in the two treatment groups. Non-parametric techniques were used for both analyses as data of interest did not follow a normal distribution.

**The sample size** was derived by estimating a reduction in the rate of flares of AD over the study period of about 20% in the placebo and 45% in the treated group, i.e. a difference between groups of 25%. The statistical significance was set for an α = 0.05 for the primary variable and the power (1-β) was set to 90%. A minimum of 84 evaluable patients per treatment group were required to show a global superiority of OM-85 over placebo assuming around 20% of drop-out rate. An exact sample size determination would require a precise estimate of the rate, which was unavailable at the time. *P*<0.05 was considered as statistically significant. Analyses were performed using SAS version 9.2 (SAS Institute) statistical software.

## Results

### Patients flow through the study

Nine of the 179 patients included did not perform visit 2 (30 days following randomization), leaving an evaluable population of 170 patients (88 to OM-85 and 82 to placebo) with at least one assessment after randomization.

**Fig 1:**
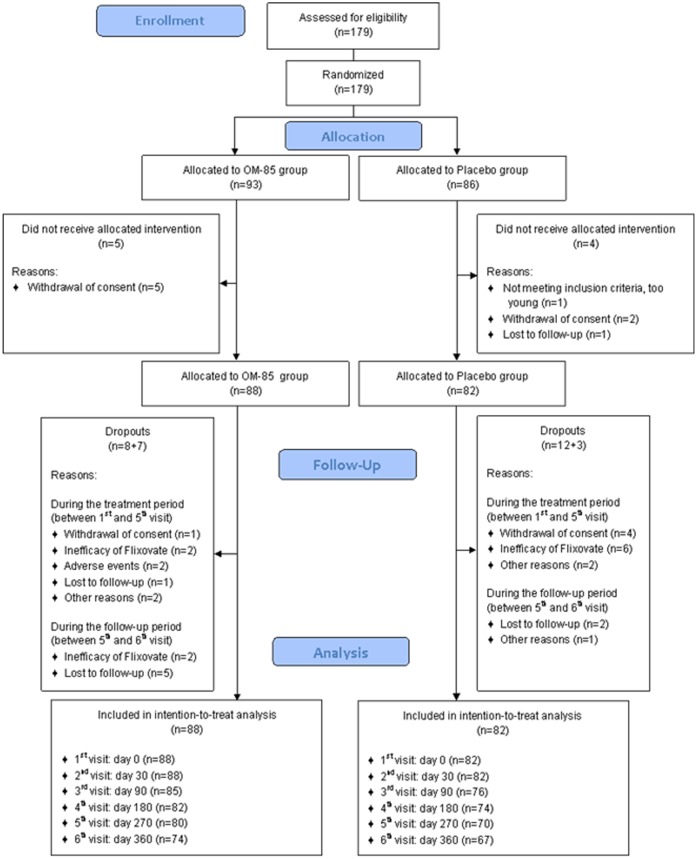
Study flowchart.

Further 19 participants withdrew during the trial, leaving a sample of 151 patients evaluable at visit 5 (270 days), i.e., the last visit during the treatment period. Ten participants withdrew between visit 5 and visit 6 (360 days), i.e., the last study visit after 3 months without study treatment (Fig 1).

### Demographic characteristics, clinical parameters at entry, and follow-up description

Between June 2003 and June 2006, 179 patients were enrolled. One hundred thirty two patients (74%) were recruited in 8 hospitals centers (67 OM-85 and 65 placebo) and 47 (26%) patients (26 OM-85 and 21 placebo) by 14 private practitioners.

No difference between groups was found with reference to demographic and clinical characteristics at entry (S1 Table). Seventy two percent and 76% of patients were recruited in hospitals centers in the OM-85 and placebo group, respectively. The median age was about 2 in both groups, with 67% and 71% of children aged 3 years or less in OM-85 and placebo group, respectively. The proportion of males was higher in the placebo (71%), compared to OM-85 group (58%) but the difference was not statistically significant. The proportion of children with a family history of atopy was similar in the two groups (67% and 68% in OM-85 and placebo group, respectively).

Concerning AD clinical evaluation, 47% and 52% of children in the OM-85 and placebo groups, respectively, reported more than 8 flares during the year prior to study entry, with about half of first symptoms occurring more than 18 months before trial inclusion in both groups. Global evaluation of AD intensity, assessed at the baseline visit, by parents was similar in OM-85 and placebo, with 44% and 34% of parents who judged the AD of their children important in the OM-85 and placebo group, respectively. According to the investigators assessment, only about 25% of children had important AD symptoms in both groups (S1 Table).

Total follow-up time was similar between groups (p = 0.83). Time to the first flare was significantly higher in the OM-85 compared to placebo group (median days: 48.5 versus 34.0, p<0.01) (S1 Table).

### Efficacy

#### Primary efficacy parameter

As expected from data reported for the year before inclusion, most children in both treatment groups experienced at least 1 NF during the study period (i.e., 75 (85%) patients in the OM-85 group and 72 (88%) in the placebo group). Overall 268 NFs of any type (regular visit (n = 71), intermediate visit (n = 45), between visit (n = 152)) were recorded in the OM-85 group *versus* 292 NFs in the placebo group (regular visit (n = 98), intermediate visit (n = 32), between visit (n = 162)). Comparable results were obtained when the mean rate of NF per patient per month (mR/_patient-month_) were considered, with significantly lower values in the patients treated with OM-85 compared to placebo (mR/_patient-month_ = 0.35, 95%CI:0.29–0.40, and mR/_patient-month_ = 0.46, 95%CI:0.40–0.52, respectively; multivariable ANOVA p-value = 0.012).

Table 2 presents the number of patients who experienced an event (NF) with the corresponding average time to event (in days) according to event order and treatment group. Two HRs estimates, and their 95% confidence intervals (CI), are reported: the first considered the multiple-events model, the second considered only the time to the first event. The HR estimate obtained from the multiple-events model for the recurrence of new events was equal to 0.80 (95% CI, 0.67–0.96) in the crude analysis and to 0.82 (95% CI, 0.69–0.98) in the multivariable model, when family history of atopy and total corticosteroids use were considered as co-variates. The first NF occurred on average 18 days later in the OM-85 group compared to placebo, and the delay was maintained over the study period for most of the subsequent events (i.e., for the 3rd, 5th, 6th and 8^th^ event), leading to fewer recurrent events per patients in the OM-85 group compared to placebo. The adjusted HR estimate for the first event was 0.70 (95% CI: 0.50–0.97). Results remain similar even when the potential confounding effect of previous number of flares was taken into account (HR = 0.82, 95% CI: 0.69–0.98, p = 0.032 and HR = 0.71 95% CI: 0.51–0.98, p = 0.040, from the multiple-events model and the first event, respectively).

**Table 2:** Repeated events (NF) description: Number of patients experiencing an event (NF), average time to event (in days) in treatment groups. HRs estimates are reported, considering both, a multiple-events model, and the time to the first event only.

	OM-85 (N = 88)		PLACEBO (N = 82)		Multiple Events (NFs) Model		first Event (NF) Model	
# of NF	Number of patients who experienced 1 to 8 events	Average time to event	Number of patients who experienced 1 to 8 events	Average time to event	HR^a^ (95% CI)	HR^b^ (95% CI)	HR^a^ (95% CI)	HR^b^ (95% CI)
**1**^**st**^	75 (85%)	60.6	72 (88%)	42.2	0.80 (0.67–0.96)	0.82 (0.69–0.98)	0.71 (0.51–0.98)	0.70 (0.50–0.97)
							*p = 0*.*039*	*p = 0*.*039*
**2** ^**nd**^	61 (69%)	52.4	64 (78%)	57.3				
**3** ^**rd**^	52 (59%)	51.8	54 (66%)	50.5				
**4** ^**th**^	39 (44%)	53.1	38 (46%)	56.9				
**5** ^**th**^	25 (28%)	48.3	30 (37%)	46.2	*p = 0*.*015*	*p = 0*.*025*		
**6** ^**th**^	8 (9%)	44.5	18 (22%)	36.4				
**7** ^**th**^	6 (7%)	37.8	11 (13%)	39.5				
**8** ^**th**^	2 (3%)	33.5	5 (6%)	24.2				

^a^ Crude analysis.

^b^ Adjusted for age, sex, history of atopy in parents, and total corticosteroids use.

In order to verify the absence of a significant bias related to potential differences between flare types (as described in detail in the Methods section, NFs could be diagnosed during regular or intermediate visits or reported by parents when they occurred between visits), we conducted a per-protocol analysis limited to children from whom data were recovered from the four follow-up visits planned by protocol (i.e. 70 in the placebo and 80 in OM-85 group respectively), and to NFs diagnosed during these planned visits only. Results were similar to those reported for the main analysis (even if HR estimates were not significant due to the smaller number of patients and events considered in this analysis). Forty nine percent of patients (N = 39) in the OM-85 group compared to 69% (N = 48) in the placebo group experienced at least one flare diagnosed during planned by protocol visits The difference in favour of OM-85 persisted for the subsequent events leading to an HR estimate by the multiple-events model of 0.77 (95% CI: 0.55–1.07) (Table 3).

**Table 3:** Repeated events (NF) description according to per-protocol analysis: Number of patients experiencing an event (NF), average time to event (in days) in treatment groups. HRs consider both a multiple-events model, and the time to the first event only.

Subgroup of patients followed up visit 5 (9 months of treatment)								
	OM-85 (N = 80)		PLACEBO (N = 70)		Multiple Events (NFs) Model		first Event (NF) Model	
# of NF	Number of Patients who experienced 1 to 4 events	Average time to event	Number of Patients who experienced 1 to 4 events	Average time to event	HR^a^ (95% CI)	HR^b^ (95% CI)	HR^a^ (95% CI)	HR^b^ (95% CI)
**1** ^**st**^	39 (49%)	85	48 (69%)	61	0.74 (0.53–1.03)	0.77 (0.55–1.07)	0.69 (0.46–1.06)	0.72 (0.47–1.09)
							*p = 0*.*091*	*p = 0*.*126*
**2** ^**nd**^	21 (26%)	128	30 (43%)	153				
**3** ^**rd**^	7 (9%)	193	15 (21%)	222	*p = 0*.*072*	*p = 0*.*123*		
**4** ^**th**^	4 (5%)	270	3 (4%)	270				

^a^ Crude analysis.

^b^ Adjusted for age, sex, history of atopy in parents, and total corticosteroids use.

When patients were stratified into subgroups according to sex, age, and family history of atopy, and SCORAD at entry (<40 and ≥40) as proxy of allergic status, the OM-85 group showed better results than the placebo group (the HRs were all below unity, even if not significant due to the low number of subjects in each strata) (S2 Table).

No significant difference emerged between groups in the number of flares registered during the last 3 months of follow-up without treatment (median in the OM-85 and placebo groups equal to 74 and 67, respectively, p = 0.73; and data not shown).

#### Secondary efficacy parameters

**Fig 2:**
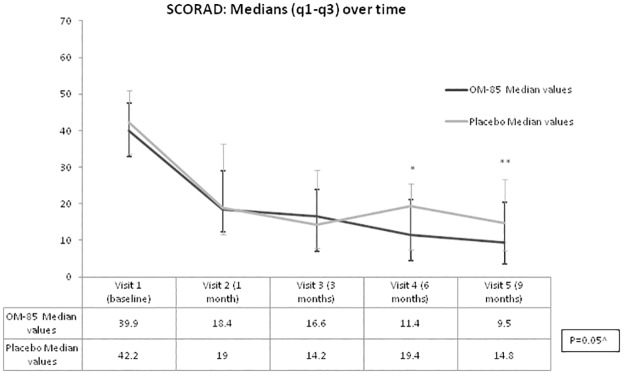
SCORAD evolution over time (median, q1 and q3) by group and visit: Visit 1 (baseline), visit 2 (1 month), visit3 (3 moths), visit 4 (6 months) and visit 5 (9 month). q1 = first quartile (25° percentile); q3 = third quartile (75° percentile). ^ p for non parametric analysis of variance (ANOVA) for repeated measures, adjusting for age and sex. * p for visit 4 = 0.02. ** p for visit 5 = 0.08.

The difference between the OM-85 and the placebo group in SCORAD evolution over time was of borderline significance (p = 0.05) (Fig 2). SCORAD was significantly lower in the OM-85 group after 6 months of treatment (p = 0.02), whereas the difference was still present but not significant after 9 months (p = 0.08).

The total consumption of corticosteroids (Flixovate^®^) was similar in the OM-85 (median = 43.0 grams, range interquartile = 20.2–91.6 grams) and the placebo (median = 42.8 grams, range interquartile = 15.7–92.4 grams) groups (p = 0.70 for non-parametric ANOVA adjusted for age and sex, data not shown).

#### Compliance and tolerability

The average global compliance over the 9-month study period as a percentage of the theoretical consumption of 1 capsule per day was satisfactory and not statistically different between groups: 90.3% (Standard deviation (SD) = 16.9) for OM-85 and 91.9% (SD = 16.9) for placebo (p = 0.38, Mann-Whitney).

One hundred fifty eight patients (82 OM-85 and 76 placebo) reported 1163 adverse events (AE) of different origin, nature, severity and duration noticed during the study (S3 Table). Among these 1163 AEs, 983 occurred during the treatment period and 180 during the follow-up period. The mean number of AEs per patient during the treatment period was similar for both groups, i.e., 5.7 (SD = 3.6) events for OM-85 and 5.8 (SD = 3.8) for placebo. The majority of these AEs were related to respiratory (54.8%) and gastrointestinal disorders (15.1%), with no significant difference between OM-85 and placebo groups. Only 5 events of the 983 that occurred during the treatment period were reported as possibly related to OM-85 (n = 3) or placebo (n = 2) (S3 Table).

## Discussion

A favourable effect of the bacterial lysate OM-85 on established AD, in particular on the recurrence of new events was reported. A protection of about 20% versus placebo (standard treatment only), in the occurrence of NFs was observed, in the absence of appreciable side effects. These results were consistent when the per-protocol group of children was considered.

These results cannot be compared side by side to those obtained with probiotics because, as mentioned above, probiotics have not been largely used in children with established AD. They may, however, be compared to those using killed bacteria or other bacterial extracts. Studies using killed *Mycobacterium vaccae* were not conclusive [28, 30, 31, 44, 45]. A first trial suggested favorable results but on a limited number of cases [44]; these results were not confirmed in a subsequent study performed by the same group conducted in younger children (2–6 years of age) [28] and also by other groups [30, 31]. *Mycobacteria vaccae* was administered parenterally (subcutaneously), in a limited number of injections, therefore in very different conditions compared to our protocol that was based on the oral administration for several consecutive months of OM-85. Modest results were published following oral administration of a bacterial lysate containing *E*. *coli* and *Enterococcus faecalis* for AD prevention and including only children with allergic heredity [46]. No efficacy was observed when the child had two allergic parents, suggesting that when heredity was “strong” the course of disease could less easily be influenced.

Our results are the first to indicate a clinical efficacy and long-term tolerability of OM-85, an oral bacterial extract, as adjuvant therapy in children with established AD, and for which the significant effect persisted even after major confounders (as corticosteroids use and AD family history) were considered. The adjuvant therapy improved the clinical outcomes of the conventional treatment with corticosteroids of 20%, which must encourage the search for clinical or biological markers to more precisely define the subgroups of responder patients.

In addition, the analysis of subgroups of patients showed the consistency of the overall results, in the absence of significant heterogeneity across strata.

These promising results suggest a possible wider use of OM-85 in the treatment of established AD. It is essential, of course, to provide more definite proof by performing further clinical trials, favouring the recruitment of children with moderate forms of AD with familiar history of atopy. Further work is also needed to analyse potential differences between different bacterial lysate preparations. Our results suggest that a mixture of bacterial derivatives might be more effective than a particulate fraction or two bacterial strains in the treatment of moderate AD [46]. Last but not least, the characterization of suitable immune biomarkers to monitor for treatment effectiveness would represent an invaluable tool to distinguish patient’s subgroups presenting differential response to treatment.

Furthermore, our data together with the results obtained with probiotics, which, as already mentioned, gave rise to a meta-analysis describing a positive result for preventive treatment [23], indicate that non-specific modulation of the immune system by bacteria antigens, both, pathogens and commensal, contributes to reduce AD occurrence and progression, which is in agreement with the Hygiene Hypothesis.

These therapeutic effects are complementary to the wealth of epidemiological data supporting the Hygiene Hypothesis [5, 47, 48]. The underlying cellular and molecular mechanisms remain however to be defined, notably the precise nature of the bacterial components involved in the protective effect (i.e., pathogens recognition receptors (PRR) ligands binding to common motifs shared by pathogenic and commensal bacteria). Another area of research could be the possible interaction of these orally administered bacterial lysates with the gut microbiome, whose diversity has been shown to correlate with AD occurrence [49–51].

## Supporting information

S1 Checklist Clinical trial checklist. (DOC)

S1 Text Study protocol. (PDF)

S1 Table Enrolment, patients baseline and follow-up characteristics, included Atopic Dermatitis (AD) clinical evaluation, by treatment group. (DOCX)

S2 Table Hazard Ratios (HR) estimates for treatment effect, obtained applying the multiple-events model (and their 95% Confidence Intervals (CI)), in subgroups of patients monitored up to the last follow-up treatment visit (9 months after randomization). (Q test p value for heterogeneity between HR estimates was showed for subgroups).
(DOCX)

S3 Table Distribution of adverse events (and concomitant diseases) as percentages of events and list of adverse events with probable/possible relationship with OM-85 or placebo. (DOCX)
