# Supplementary material for: Adjuvant treatment with the bacterial lysate (OM-85) improves management of atopic dermatitis: A randomized study
Source: PLoS One. 2017 Mar 23;12(3):e0161555. doi: 10.1371/journal.pone.0161555 (PMC5363804; doi:10.1371/journal.pone.0161555)
Supplement: S1 Text — (PDF) [file pone.0161555.s002.pdf]

CONFIDENTIAL: This protocol is the property of OM PHARMA, Meyrin / Geneva, Switzerland. Data contained in the protocol are confidential and may not be used nor disclosed without the prior permission of the sponsor.

CLINICAL STUDY PROTOCOL

Multicentric, randomised, double blind, placebo-controlled study of the efficacy and safety of Imocur® (OM-85 BV) in children suffering from atopic dermatitis.

Study Reference Number:	BV-2002/1
Development Phase :	III
Date of Final Protocol :	22/11/2002
Study Co-ordinators	Pr Christine Bodemer<br>Service de Dermatologie<br>Hôpital Necker-Enfants Malades<br>149 Rue de Sèvres 75015 Paris<br>France
Sponsor :	OM PHARMA<br>22, rue du Bois-du-Lan<br>CH-1217 Meyrin 2 / Geneva<br>Switzerland

Number of pages: 65

STUDY PERSONNEL

**Study Co-ordinator :**	**Pr Yves De Prost**<br>**Pr Christine Bodemer**<br>Service de Dermatologie<br>Hôpital Necker-Enfants Malades<br>149 Rue de Sèvres 75015 Paris, France
**Co-Investigator(s) :**	The list of Co-Investigators will be provided separately
**Sponsor :**	**OM PHARMA**<br>22, rue du Bois-du-Lan<br>CH-1217 Meyrin 2 / Geneva<br>Phone: + 41/22 783 11 11<br>Fax: +41/22 783 11 22
**Clinical Study Responsible :**	**Mr. Stéphane Deshormières**<br>Clinical Research Associate<br>OM PHARMA<br>22, rue du Bois-du-Lan<br>CH-1217 Meyrin 2 / Geneva<br>Phone: + 41 22 783 14 91<br>Fax: +41 22 783 14 15<br>e-mail: stephane.deshormieres@ompharma.com
**Pharmacovigilance Officer:**	**Dr. Gabriel Cozma**<br>Head of Medical & Regulatory Affairs<br>OM PHARMA<br>22, rue du Bois-du-Lan<br>CH-1217 Meyrin 2 / Geneva<br>Phone: + 41 22 783 14 71<br>Fax: +41 22 783 14 15<br>e-mail: gabriel.cozma@ompharma.com
**Biostatistician Advisor :**	**Mrs Liliane Marie Chatenoud**<br>Via Martiri Triestini 10, 20148 Milan, Italy<br>Phone: 0039 02 39014577<br>e-mail: Liliana@marionegri.it
**CRO :**	**Dr Urs Billeter**<br>Medical Director, Pharma Part AG<br>Bahnhofstrasse 20, Postfach 171<br>CH-8800 Thalwil, Switzerland<br>Phone: + 41 22 723 59 27<br>e-mail: UrsB@pharmapart.com

SYNOPSIS

**Sponsor:**	OM PHARMA, 22, rue du Bois-du-Lan, CH-1217 Meyrin 2 / Geneva
**Title of the study:**	Multicentric, randomised, double blind, placebo-controlled study of the efficacy and safety of Imocur® (OM-85 BV) in children suffering from atopic dermatitis.
**Study reference number:**	BV-2002/1
**Study co-ordinators:**	Professors Yves de Prost and Christine Bodemer
**Indication:**	Atopic dermatitis (AD)
**Study phase:**	III
**Study objectives:**	To evaluate the efficacy and safety of Imocur 1 capsule (3.5mg) per day for 9 months compared to placebo on the evolution of the disease in children suffering from atopic dermatitis.
**Study design:**	Multicentric, randomised, double-blind, placebo-controlled
**Study centres:**	8 study centres in France.
**Number of patients:**	208 patients to be enrolled (based on sample size calculation for assumed superiority with N = 168).
**Main inclusion criteria:**	Children outpatients of both sexes, aged 6 months to 7 years, with atopic dermatitis confirmed by Haniffin-Rajka or Williams *et al*, and a SCORAD between 25 and 70.
**Main exclusion criteria:**	Children under general corticotherapy within one month of study start, patients with immunodeficiency, patient's affected body surface area less than 15% or greater than 70% or with known allergy to desonide.
**Treatment:**	The drug regimen will be 1 capsule (3.5 mg) of Imocur or placebo per day during 9 months, starting at inclusion.
**Time schedule:**	Screening visit on day of start of trial treatment, then 4 follow-up visits after respectively 1, 3, 6, 9 months of treatment and 1 post-treatment visit 12 months after inclusion.
**Primary parameter of efficacy:**	Comparisons between the two groups of the number of AD crisis during the study starting after 1 month of treatment.
**Secondary parameter of efficacy:**	Comparisons between the two groups based on SCORAD evaluation, amount of corticoids used and parents/investigator assessment.
**Safety parameters:**	Assessments of safety will consist of measurements of vital signs, performance of physical examinations and monitoring of adverse events and serious adverse events
**Statistical analysis:**	An intent-to-treat analysis will be performed, *i.e.* all randomised subjects, regardless of their compliance with the protocol.<br>The primary efficacy parameter will be the rate of AD-'Crisis' (cumulated per patient, per month) computed at each visit until the final visit. It will be the only parameter used for comparing the efficacy of OM-85 to that of placebo. AD crisis rate is intended to be tested by means of non parametric analysis considering also potential covariates (such as corticosteroid use).<br>The interim safety and efficacy results of this trial will be monitored independently. There will be one formal interim analysis after 80 patients will have reached the 5th visit (9 months of inclusion into the trial).
**Study timelines:**	Expected start date (first patient – first visit): 04/2003<br>Expected end date (last patient - last visit): 04/2005<br>Expected completion of Clinical Study Report: 04/2006

FLOW CHART

Visit Number	1	2	3	4	5	6[1]	A, B, etc.
**Week / Month**	**Day 0 Screening**	**Month 1 ± 7 days**	**Month 3 ± 7 days**	**Month 6 ± 7 days**	**Month 9 ± 7 days**	**Month 12 ± 7 days**	**Intermed.**
**Informed consent**	X						
**Demographic data**	X						
**Past and concomitant disease(s)**	X						
**Inclusion - & exclusion criteria**	X						
**Concomitant medications**	X	X	X	X	X	X	X
**Clinical status/vital signs**	X	X	X	X	X	X	X
**History of the disease**	X						
**Randomisation**	X						
**Drug dispensing**	X	X	X	X			
**Additional prescription**	X	X	X	X	X		X[2]
**Diary card dispensing**	X	X	X	X	X		
**Study medication compliance**		X	X	X	X		
**SCORAD evaluation**	X	X	X	X	X	X	X
**Affected body surface area**	X	X	X	X	X	X	X
**Accounting of dermocorticoids**		X	X	X	X	X	
**Investigators / parents assessment**	X	X	X	X	X	X	X
**Adverse events**		X	X	X	X	X	X
**Accounting of AD flare**	X	X	X	X	X	X	X
**End of trial**						X	

[1] Or at premature discontinuation

[2] If applicable

TABLE OF CONTENTS

STUDY PERSONNEL 2
SYNOPSIS 3
FLOW CHART 4
1 BACKGROUND AND RATIONALE FOR THE STUDY 8
2 OBJECTIVE OF THE STUDY AND ENDPOINTS 10
2.1 OBJECTIVE OF THE STUDY 10
2.2 PRIMARY ENDPOINT 10
2.3 SECONDARY ENDPOINT(S) 11
2.4 SAFETY 11
3 STUDY DESIGN 11
4 STUDY POPULATION 12
4.1 INCLUSION CRITERIA 12
4.2 EXCLUSION CRITERIA 12
4.3 WITHDRAWAL CRITERIA 13
5 STUDY MEDICATION 13
5.1 INVESTIGATIONAL DRUG 13
5.2 COMPARATOR DRUG 13
5.3 DOSAGE AND TREATMENT SCHEDULE 13
5.4 SUPPLY, LABELLING AND PACKAGING ARRANGEMENTS 14
5.5 METHODS OF BIAS REDUCTION 14
5.5.1 RANDOMISATION 14
5.5.2 BLINDING 15
5.5.3 EMERGENCY CODE 15
5.6 TREATMENT ASSIGNEMENT 15
5.7 STORAGE CONDITIONS 15
5.8 CONCOMITANT MEDICATIONS 15
5.8.1 AUTHORIZED MEDICATIONS INCLUDING RESCUE MEDICATION 15
5.8.2 UNAUTHORIZED MEDICATIONS 16
6 STUDY PROCEDURES AND ASSESSMENTS 16
6.1 STUDY PROCEDURE 16
6.1.1 RECRUITMENT PROCEDURE 16
6.1.2 DEMOGRAPHIC DATA, SCREENING DATA 16

6.1.3 VISIT SCHEDULE.......16
6.2 STUDY PROCEDURES AT EACH VISIT.......17
6.2.1 VISIT 1 (V1), SCREENING, MONTH 0.......17
6.2.2 VISIT 2 (V2), MONTH 1 (±7 DAYS).......17
6.2.3 VISIT 3 (V3), MONTH 3 (±7 DAYS).......18
6.2.4 VISIT 4 (V4), MONTH 6 (±7 DAYS).......18
6.2.5 VISIT 5 (V5), MONTH 9 (±7 DAYS).......19
6.2.6 VISIT 6 (V6), MONTH 12 (±7 DAYS) OR AT PREMATURE DISCONTINUATION.......19
6.2.7 INTERMEDIARY VISITS, VISITS A, B, ETC.......19
6.3 REPLACEMENT OF DROP-OUTS AND WITHDRAWALS.......20
7 ASSESSMENT OF EFFICACY AND SAFETY.......20
7.1 ASSESSMENT OF EFFICACY.......20
7.1.1 PRIMARY EFFICACY PARAMETER.......20
7.1.2 SECONDARY EFFICACY PARAMETER(S).......20
7.2 ASSESSMENT OF SAFETY.......21
7.3 LABORATORY ASSESSMENTS.......21
7.4 ASSESSMENT OF COMPLIANCE.......21
8 ADVERSE EVENTS.......21
8.1 REPORTING AND RECORDING OF ADVERSE EVENTS.......22
8.2 REPORTING, RECORDING AND HANDLING OF SERIOUS ADVERSE EVENTS (SAES).......23
9 DATA MANAGEMENT AND STATISTICS.......23
9.1 DATA MANAGEMENT.......23
9.2 BLIND REVIEW.......23
9.3 SAMPLE SIZE CALCULATION.......24
9.4 CONFIRMATORY ANALYSIS OF EFFICACY CRITERIA.......24
9.5 EXPLORATORY ANALYSIS OF EFFICACY CRITERIA.......25
9.6 ANALYSIS OF SAFETY DATA.......25
9.7 ANALYSED POPULATION.......25
9.8 DATA ANALYSIS AND COMPUTER PROGRAM.......26
9.9 INTERIM ANALYSIS.......26
10 ETHICAL CONSIDERATIONS AND PATIENT'S CONSENT.......26
10.1 ETHICAL CONSIDERATIONS.......26
10.2 ETHICS COMITTEES (EC), NATIONAL REGULATORY AUTHORITIES.......26
10.3 INFORMED CONSENT.......27
11 INSURANCE.......27

12 QUALITY ASSURANCE / AUDIT.....27
13 PATIENT DOCUMENTATION AND MONITORING.....28
13.1 CASE REPORT FORM (CRF).....28
13.2 MONITORING.....28
14 DRUG ACCOUNTABILITY.....29
15 ADMINISTRATIVE ISSUES.....29
15.1 RULES FOR AMENDING THE PROTOCOL.....29
15.2 DISCONTINUATION OF THE TRIAL BY THE SPONSOR.....29
15.3 ARCHIVING.....29
16 STATEMENT OF CONFIDENTIALITY.....29
17 SECRECY AGREEMENT.....30
18 REPORTING AND PUBLICATION.....30
18.1 REPORTING.....30
18.2 PUBLICATION.....30
19 FINANCIALS.....30
20 CONTINUATION SUPPLY.....30
21 SCIENTIFIC AGREEMENT / SIGNATURES.....31
21.1 AGREEMENT.....31
21.2 SIGNATURES.....31
22 REFERENCES.....33
23 ATTACHMENTS TO THE PROTOCOL.....35
23.1 SCORAD INDEX.....36
23.2 THE CRITERIA OF HANIFIN AND RAJKA.....39
23.3 WRITTEN PATIENT INFORMATION SHEET.....43
23.4 SAMPLE OF INFORMED CONSENT FORM.....48
23.5 INSURANCE CERTIFICATE.....49
23.6 SERIOUS ADVERSE EVENT REPORT FORM (FOR SAE REPORTING).....51
23.7 DECLARATION OF HELSINKI.....55
23.8 LABELLING.....60
23.9 OBLIGATIONS OF SPONSOR AND INVESTIGATOR.....63

1 BACKGROUND AND RATIONALE FOR THE STUDY

Atopic dermatitis (AD) is a chronic and relapsing inflammatory skin disease with immunological disturbance, and its incidence is increasing in infants and children (1). Patients with AD have been reported to have typical eczema with pruritus, most frequently accompanied by increased levels of total and/or specific IgE in the peripheral blood, and a personal or familial history of atopic diseases (2). In addition, immunological disturbance towards a Th2 like phenotype has been noticed in patients with AD, such as overexpression of IL-3, IL-4, IL-5, IL-10 and granulocyte-macrophage CSF in the affected skin (3,4). However, the mechanisms of IgE hyperproduction in the pathogenesis of AD have not been clarified.

It is one of the first manifestations of allergy in early childhood, starting in infants during the first months of life, generally around 3 months but sometimes even earlier.

- **Clinical features**

Atopic dermatitis remains a clinical diagnosis. The aspect of the lesions varies according to the severity of AD and the time of evaluation (during crisis or remittance). Major diagnostic criteria are personal or family history of atopy (AD, rhinitis, allergic conjunctivitis, allergic blepharitis, asthma), morphology and distribution of the lesions, pruritus and chronic or recurrent dermatosis.

Pruritus is a consistent feature of AD and cutaneous signs can change depending on the age of the lesions. Acute lesions of AD are eczematous-erythematous, scaling, papulovesicles and oozing and crusted lesions may appear as well.

During recurrences of this inflammatory dermatosis, different stages of severity can be observed, from one crisis to the other in a given child, and from one child to the other.

Clinical expression of AD also changes with the patient’s age. The infantile stage occurs up to approximately 2 years. 90% of AD occur before the fifth year and 60% during the first year of life.

Most physicians involved in the care of patients with atopic dermatitis rely on the diagnostic criteria of Hanifin and Rajka (5) or Williams et al. (6). However assessment methods are far from being standardised, and therapeutic studies are often difficult to interpret. Severity criteria can be precisely defined using different types of quotation, in particular SCORAD (for **SCOR**ing **A**topic **D**ermatitis) (6), which takes into account the characteristics of the initial lesion: erythema, edema, excoriation, oozing, and characteristic pruritus intensity, as well as affected body surface area. Maximum SCORAD for extremely severe AD is 103. For research purpose, SCORAD offers a more detailed and comprehensive assessment (7).

At present, no definitive treatment exists for this chronic inflammatory dermatosis starting in primary childhood and potentially lasting until adulthood. The more classical reference treatment is a treatment with dermocorticoids prescribed if justified by the inflammatory crisis.

Dermocorticoids are used to treat the disease crisis. They only display a symptomatic effect, they do not avoid disease recurrence and their long-term use may trigger well known side effects, which can cause important problems in children and particularly in infants.

- **Investigational drug**

Imocur® (also called Broncho-Vaxom®), an orally administered immunostimulating preparation, is a lyophilised bacterial extract prepared from 8 bacterial species *(Haemophilus influenzae, Streptococcus pneumoniae, Klebsiella pneumoniae and ozaenae, Staphylococcus aureus, Streptococcus pyogenes and viridans, Neisseria catarrhalis).*

A variety of *in vitro* pharmacodynamic studies give evidence that Imocur® acts primarily on different functions of the non-specific immune defence mechanisms, rather than provoking specific immune responses to the various bacterial antigens it contains. *In vitro,* it has been demonstrated that Imocur® increases the secretion of TNF-α and IL-2 in cultured macrophages (8). The metabolic (production of toxic oxygen metabolites) and functional (lytic) activities in murine macrophages are increased by Imocur® (9) as well as the antigen processing (10). The expression of certain adhesion molecules is upregulated in human PMNs and monocytes (11).

It has been shown that the secretion of salivary sIgA was significantly increased in humans after Imocur® administration (12) and different parameters in the broncho-alveolar lavage fluid were influenced: γ-interferon and macrophage activity were increased and the T helper/T suppressor ratio was modified (13,14).

The efficacy of Imocur® has been investigated in numerous clinical studies, both in adults and children.

Martin du Pan (15), Ahrens (16), Zagar (17) and Maestroni (18) investigated a total of over 300 children presenting with infections of the upper respiratory tract in placebo-controlled, double-blind clinical trials.

The main objective of all these studies was to evaluate the preventive effect of Imocur® on recurrent respiratory infections. One author (17) also evaluated the possible short-term therapeutic benefit of Imocur® during the acute phase of chronic rhinosinusitis.

Imocur® has been shown to present a good clinical safety and is already used in children and even in infants over 6 months in several countries, in the prevention of chronic pulmonary diseases.

- **Rationale for the use of Imocur® in the treatment of atopic dermatitis**

It is notably known, particularly from the work of Holt, that children at birth are Th2 oriented and that the shift should be pushed towards Th1 in order to avoid allergy later on (19,20,21,22). Imocur® was shown to induce an immunomodulation by a non-specific immunostimulation.

Bowman and Holt (23) and Bessler (24) showed that Imocur® was able to restore the Th1/Th2 balance towards Th1, a mechanism of action compatible with its beneficial effects observed in the struggle against infections.

Other clinical and laboratory studies (25,26,27) have shown OM-85-BV to increase the resistance to respiratory tract infections, to possess immunomodulatory activities on T cells and a regulatory action on immunoglobulins, particularly a reduction of high IgE levels (28,29).

The potential protective effect on early infections upon appearance of AD has recently been mentioned, in parallel with epidemiology studies showing an increase of AD prevalence (between 10 and 20% in the pediatric group) during the last 20 years. This increase is much more important in Western countries and populations of high social class than in Eastern countries or with a lower standard of living. Moreover, the prevalence of AD is lower in large families and in children, who are rapidly attending day care nurseries. There might be a protective effect against infection in the first months of life, regardless of the viral, bacterial or parasitic origin of the infection. The hypothesis is that infections inhibit IgE production by stimulating TH1 cells. They thus inhibit clonal expansion of TH2 cells and therefore modify the TH1/TH2 balance.

Moreover, intradermal injections of *Mycobacterium vaccae* have been shown recently to be associated with an improvement of children with moderate to severe AD. Administration of PROBIOTICS to pregnant women before and after delivery was shown to decrease the early development of atopic dermatitis by high-risk children.

These data suggest that such treatment as Imocur, by inducing an early contact with bacterial extracts, could modulate the immunity of children with AD, and thus play an active role in the treatment of this pathology.

The trial will be conducted following the GCP guidelines. The trial is intended to support a new registration of the product in this indication.

2 OBJECTIVE OF THE STUDY AND ENDPOINTS

2.1 OBJECTIVE OF THE STUDY

The objective of this pilot double-blind study is to evaluate the efficacy and the safety of Imocur® 1 capsule (3.5 mg) per day for 9 months compared to placebo on the evolution of the disease in children suffering from atopic dermatitis.

2.2 PRIMARY ENDPOINT

The primary efficacy parameter is:

- Comparison of the number of AD flare during the course of the study, starting evaluation after 1 month of treatment (baseline) and during the study period, between the two groups. For this comparison, the scale detailed below will be used. To define a new flare, a period of 15 days since the last flare is required.

A score of the atopic dermatitis flare will be attributed based on this scale.

Score	Definition
0 = remission	No signs of cutaneous inflammatory
1 = close to remission	Very slight erythema, and/or infiltration just perceptible.
2 = minor lesions	Erythema visible but small and/or slight infiltration/papules
3 = mild lesions	Erythema of mild intensity, infiltration/papules of mild intensity. Pruritus, crusted lesions.
4 = severe lesions	Marked erythema and/or infiltration/papules precise, pruritus, crusted lesions.
5 = highly severe lesions	Intense erythema, infiltration/papule marked with oozing/excoriation.

An AD flare is defined as: recurrence of AD lesions quoted at least 3 according to global evaluation criteria with a minimum of 15 days from the last flare.

2.3 SECONDARY ENDPOINT(S)

The secondary efficacy parameters are:

- SCORAD: (see attachment 23.1)

  Comparison of SCORAD at Month 1 (considered as baseline status) and Month 3 / Month 6 / Month 9 between the 2 groups.

- Affected body area:

  Comparison of affected body surface area at Month 1 (considered as baseline status) and Month 3 / Month 6 / Month 9 between the 2 groups.

- Consumption of topical corticosteroid during the study:

  This parameter will be assessed by counting and weighing the tubes prescribed, used and brought back by the families.

- Assessment by the parents/children and by the investigator

  Assessment of the global improvement of the dermatosis by answering the question "how do you judge the improvement of atopic dermatitis in the child since the beginning of the study?» (comment jugez-vous de l'amélioration de la dermatite atopique de l'enfant depuis le début de l'étude?). The answer will be put on a scale graded with 5 items as follows: 0=null (nulle), 1=mild (peu importante), 2=moderate (modérée), 3=important (importante), 4=very important (très importante).

2.4 SAFETY

The safety and tolerability of Imocur® will be evaluated by monitoring vital signs and the occurrence of adverse events.

3 STUDY DESIGN

The study will be a multicentric, randomised, double blind, placebo-controlled phase III study.

208 children aged 6 months to 7 years with atopic dermatitis will be recruited in 8 centres in France in order to achieve 168 evaluable cases for the statistical analysis.

'Evaluable' means having performed all six scheduled clinical visits, with a good compliance (80% of study drug intake).

In order to avoid secondary evaluation bias due to seasonal changes, the inclusion period will last one year, from March 2003 until February 2004.

Children will be followed over a period of 1 year, including 9 months of treatment.

The end of the clinical part will therefore be around December 2004.

4 STUDY POPULATION

After written informed consent will have been obtained from the parents (and children if applicable), children of both sexes, aged 6 months to 7 years (in their eighth year of life), suffering from atopic dermatitis will be included in the trial.

4.1 INCLUSION CRITERIA

- Male or female children aged 6 months to 7 years (in their eighth year of life);
- Children with atopic dermatitis confirmed by Hanifin-Rajka (see attachment 23.2) or Williams et al (see attachment 23.3) with affected body surface area ≥ 15% and ≤ 70%;
- 25 ≤ SCORAD ≤ 70;
- Written informed consent obtained from the parents/legal guardian (and the child if applicable).

4.2 EXCLUSION CRITERIA

- Children under general corticotherapy within one month of study start;
- Children with immunodeficiency;
- Children with SCORAD < 25 or > 70;
- Children with affected body surface area <15% or >70%;
- Children with autoimmune disease;
- Children with malignant disease;
- Children under immunosuppressive or immunostimulating therapy within 1 month of study start;
- Children whose parents or guardians are unable to comply with the requirements of the protocol, especially if they are thought to be unable to complete the patient's diary card;
- Children with a known allergy or previous intolerance or known hypersensitivity to the trial drug or to any of the corticoids used;
- Participation in another clinical trial and/or treatment with an experimental drug within 3 months of study start and during the present trial.

4.3 WITHDRAWAL CRITERIA

- Children whose parents/legal guardian withdraw consent
- Children lost to follow-up
- Unauthorised concomitant medication
- Occurrence of serious adverse drug reactions (at least in suspected relationship with trial treatment) or of other intolerable adverse events, as judged by the investigator
- Major protocol violations
- Unsatisfactory efficacy (in the investigator's opinion)
- Patient's condition does no longer require trial treatment (in the investigator's opinion)
- Death
- Administrative reasons (e.g. subject moves and discontinues due to distance)
- Children not responding to Flixovate cream® treatment
- Other reasons

The reason for withdrawal must be reported on the page of the "Terminal Study Status" of the CRF and all study medication has to be returned. For withdrawn children, a full examination according to the last planned visit should be performed, whenever possible.

For replacement of withdrawn patients see section 6.3.

5 STUDY MEDICATION

5.1 INVESTIGATIONAL DRUG

The investigational drug supplied is Imocur® (also called Broncho-Vaxom®). Imocur® is a lyophilized extract of 8 bacterial species (*Haemophilus influenzae, Streptococcus pneumoniae, Klebsiella ozaenae and pneumoniae, Staphylococcus aureus, Streptococcus viridans and pyrogenes, Neisseria catarrhalis*). Each capsule contains 3.5 mg of lyophilised bacterial extract as active principle.

5.2 COMPARATOR DRUG

Matched placebo capsules will be used as comparative treatment.

5.3 DOSAGE AND TREATMENT SCHEDULE

The posology will be one capsule per day of either Imocur® or placebo, to be taken with a meal or with a little water. In young children who cannot swallow the whole capsule, it is recommended to open it and pour its contents into some liquid (tea, milk, juice….).

In addition to the study drug, all children included into the study will receive conventional treatment with emollient and topical corticoids. In order to harmonise treatments, the same following drugs will be prescribed to all children:

- Dexeryl® one application daily during the whole study.

- Flixovate cream® 1 application daily during 8 days followed by 1 application every 2 days during 8 days and then 1 application every 3 days during 8 days. Flixovate cream® will be prescribed at each visit. In case of persistence of the symptoms with Flixovate cream®, the patient will be withdrawn from the study.

The duration of the study treatment per patient will be 9 months, followed by a 3 month follow-up period without study treatment.

5.4 SUPPLY, LABELLING AND PACKAGING ARRANGEMENTS

Drug supplies will be provided by the sponsor. The investigator(s) will be supplied with randomised trial medication (active and placebo) for a maximum of 32 patients.

The drug supplies for one centre will be packed in a “centre box”. The “centre box” contains the adequate number of “patient kits” which contain the medication for individual patients. The “patient kit” contains the required number of “medication boxes”, each of them intended for a 1 month treatment period.

The “patient kit” containing the medication for the double-blind treatment phase of one patient will bear a randomisation number identified by the SCORAD at entry (< 40 or ≥ 40), and treatment number A001 to A128 or B001 to B128 respectively, depending on the SCORAD value.

The trial medication is presented in blisters of 10 capsules each. For each patient a total of 300 capsules (incl. 30 reserve capsules) of the active treatment or placebo will be supplied for the entire treatment duration of 9 months.

The blisters will be packed in medication boxes containing 30 capsules each (3 blisters of 10 capsules) and sufficient for the treatment during one month. Each patient will be provided with 10 medication boxes including the nine-month treatment and one box of reserve. The reserve capsules included in each medication box can be used in case of delayed attendance at the scheduled visit or in any other case judged necessary by the investigator.

The labels on the study medication will comply with French legislation; in particular, they will bear the patient randomisation number and the study reference (attachment 23.9).

The process of blind packaging is performed in the clinical pharmacy department of the sponsor company. This department receives a randomisation list for packaging purposes in a sealed envelope. The packaging is done according to special SOPs in a validated environment.

5.5 METHODS OF BIAS REDUCTION

5.5.1 RANDOMISATION

Patients will be randomly allocated to treatment groups, according to the random permuted block scheme. In order to balance both treatment arms, one factor is taken into account for the randomisation: SCORAD at entry. For each centre a balanced set of treatments is prepared. In accordance with the ICH Biostatistics Guideline, the block size is intentionally not given in the study protocol.

The generation of the random code list including the production of sealed envelopes is performed in a validated environment at the clinical pharmacy department of the sponsor company.

5.5.2 BLINDING

The study is conducted as a double-blind study. The test preparation does not differ from the placebo comparator concerning form, colour, texture of content, etc.

5.5.3 EMERGENCY CODE

The investigator will be provided with a sealed envelope containing the code for each patient's randomisation number. The envelopes will be returned to the Sponsor at the end of the study and will be checked to ensure that the seals have not been broken unnecessarily. The code can only be broken under serious adverse event circumstances. If the investigator feels a code-break is required, the monitor and/or the pharmacovigilance officer should first be consulted unless the delay would jeopardise the patient’s status.

If a code-break occurs, the investigator will record the reason for the code-break, the date of opening and will sign the envelope in the appropriate place, withdrawing the patient from the trial. The investigator will communicate the event of breaking the code to the Sponsor.

5.6 TREATMENT ASSIGNEMENT

Each patient will be assigned to one of the following medication blocks, according to his/her SCORAD upon inclusion in the study:

Block A: SCORAD < 40

Block B: SCORAD ≥ 40

In order to ensure random allocation, each new patient randomised is to be given the medication box bearing the lowest available randomisation number, in her/his respective medication block.

The trial drug will first be dispensed at visit V1 for one month. During the course of the trial, the medication will then be dispensed at visits V2 for two months and at V3 and V4 for three months respectively. Dispensed medication has been calculated to cover the period until the next scheduled visit. The reserve box will be given to each patient during the first visit in order to cope with any delayed attendance at the scheduled visit or in any other case judged necessary by the investigator.

5.7 STORAGE CONDITIONS

Drug supplies must be kept in a secure, limited access storage area at room temperature (15-25°C) away from humidity and light. Clinical supplies must be kept out of the reach of children and any person not involved in the trial.

5.8 CONCOMITANT MEDICATIONS

5.8.1 AUTHORIZED MEDICATIONS INCLUDING RESCUE MEDICATION

Concomitant medication, as long as it is not mentioned under 5.8.2. is allowed, however has to be documented in the CRF, as to International Non-proprietary Name (INN) or trademark, strength, galenic form, route, dosage, date of onset and of cessation, reason for the therapy.

The patient should not take any concomitant treatment without the investigator’s knowledge.

In particular, general antibiotherapy due to impetigo is authorised on inclusion as well as during the study.

Planned vaccinations according to the usual vaccination calendar can be performed during the study.

5.8.2 UNAUTHORIZED MEDICATIONS

The following concomitant medications that might interfere with the clinical results are prohibited during the whole study duration and during the last month before study start:

- General corticotherapy
- Immunosuppressive or immunostimulating therapy

6 STUDY PROCEDURES AND ASSESSMENTS

6.1 STUDY PROCEDURE

6.1.1 RECRUITMENT PROCEDURE

At visit 1, before any assessment necessary for the study, the parent(s)/legal guardian of the child (and the child, depending on the age) will be informed by the physician according to the patient information sheet (see attachment 23.4). The parent(s)/legal guardian (and the child if applicable) will then give a written consent by signing the "Patient Consent Form" (see attachment 23.5).

At visit 1, children will be assessed with respect to their eligibility for the trial. Children qualifying for the trial and for whom written informed consent has been obtained will be identified by their initials, sex and birth date.

At this visit the children will be assigned a letter and 3-digit randomisation number (e.g. between A001 and A128).

6.1.2 DEMOGRAPHIC DATA, SCREENING DATA

The screening visit (V1) will enclose the following parameters:

- Demographic data (initials, birth date, age, sex, ethnic origin)
- Clinical status
- Relevant previous medical case history and family history
- Past and concomitant diseases
- Clinical diagnosis of the atopic dermatitis

6.1.3 VISIT SCHEDULE

All clinical visits should conform as closely as possible to the schedule in flowchart on page 4. A window of seven days (± 7) will be acceptable at all visits.

Visits are foreseen at screening (V1), after 1 month (V2), 3 months (V3), 6 months (V4), 9 months (V5) and 12 months (V6 = Final Visit).

Intermediary visits will take place, if necessary, in case of non-improvement of the treated flare or in case of a new flare.

6.2 STUDY PROCEDURES AT EACH VISIT

6.2.1 VISIT 1 (V1), SCREENING, MONTH 0

During this screening visit, the following parameters are checked, collected and reported on the Case Report Form (CRF):

- Date of the visit
- Informed consent
- Demographic patient data (birth date, sex, ethnic origin)
- Recording of atopic dermatitis history (previous crisis) and family history
- Concomitant diseases and concomitant medication(s)
- Inclusion-, exclusion criteria
- Physical examination (i.e. clinical status at the time of examination)
- SCORAD
- Affected body surface area
- Delivery of trial medication for one month, incl. reserve medication
- Delivery of diary card, including instructions
- Prescription of Dexeryl and Flixovate cream

6.2.2 VISIT 2 (V2), MONTH 1 (±7 DAYS)

During this visit, the following parameters are checked, collected and reported on the Case Report Form (CRF):

- Date of visit
- Compliance with the protocol
- Number of intermediary visit
- Concomitant medication(s)
- Physical examination (i.e. clinical status at the time of examination)
- Accounting of AD flare
- SCORAD
- Affected body surface area
- Number of capsules returned
- Number of corticosteroids tubes used since last visit and weight
- Investigator's assessment of the efficacy
- Assessment by the parents/child of the global improvement
- Adverse events (if applicable)
- Delivery of trial medication for two months, incl. reserve medication
- Prescription of Dexeryl and Flixovate cream.

6.2.3 VISIT 3 (V3), MONTH 3 (±7 DAYS)

During this visit, the following parameters are checked, collected and reported on the Case Report Form (CRF):

- Date of visit
- Compliance with the protocol
- Number of intermediary visit
- Concomitant medication(s)
- Physical examination (i.e. clinical status at the time of examination)
- Accounting of AD flare
- SCORAD
- Affected body surface area
- Number of capsules returned
- Number of corticosteroids tubes used since last visit and weight
- Investigator’s assessment of the efficacy
- Assessment by the parents/child of the global improvement
- Adverse events (if applicable)
- Delivery of trial medication for three months, incl. reserve medication
- Prescription of Dexeryl and Flixovate cream.

6.2.4 VISIT 4 (V4), MONTH 6 (±7 DAYS)

During this visit, the following parameters are checked, collected and reported on the Case Report Form (CRF):

- Date of visit
- Compliance with the protocol
- Number of intermediary visit
- Concomitant medication(s)
- Physical examination (i.e. clinical status at the time of examination)
- Accounting of AD flare
- SCORAD
- Affected body surface area
- Number of capsules returned
- Number of corticosteroids tubes used since last visit and weight
- Investigator’s assessment of the efficacy
- Assessment by the parents/child of the global improvement
- Adverse events (if applicable)
- Delivery of trial medication for three months, incl. reserve medication
- Prescription of Dexeryl and Flixovate cream.

6.2.5 VISIT 5 (V5), MONTH 9 (±7 DAYS)

During this visit, the following parameters are checked, collected and reported on the Case Report Form (CRF):

- Date of visit
- Compliance with the protocol
- Number of intermediary visit
- Concomitant medication(s)
- Physical examination (i.e. clinical status at the time of examination)
- Accounting of AD flare
- SCORAD
- Affected body surface area
- Number of capsules returned
- Number of corticosteroids tubes used since last visit and weight
- Investigator's assessment of the efficacy
- Assessment by the parents/child of the global improvement
- Adverse events (if applicable)
- Prescription of Dexeryl and Flixovate cream.

6.2.6 VISIT 6 (V6), MONTH 12 (±7 DAYS) OR AT PREMATURE DISCONTINUATION

During this visit, the following parameters are checked, collected and reported on the Case Report Form (CRF):

- Date of visit
- Compliance with the protocol
- Number of intermediary visit
- Concomitant medication(s)
- Physical examination (i.e. clinical status at the time of examination)
- Accounting of AD flare
- SCORAD
- Affected body surface area
- Investigator's assessment of the efficacy
- Assessment by the parents/child of the global improvement
- Adverse events (if applicable)
- End of trial

6.2.7 INTERMEDIARY VISITS, VISITS A, B, ETC.

If an AD crisis occurs between two scheduled visits, the patient is asked to come back to the investigator as soon as possible. The information collected on that(these) occasion is(are) to be reported on a special section of the case report form.

During this(these) visit(s), the following parameters are checked, collected and reported on the special section of the Case Report Form (CRF):

- Date of visit
- Reason for the intermediary visit (treatment not appropriate, new flare…)
- Concomitant medication(s)
- Physical examination (i.e. clinical status at the time of examination)
- SCORAD
- Affected body surface area
- Investigator's assessment of the efficacy
- Assessment by the parents/child of the global improvement
- Adverse events (if applicable)
- Prescription of Flixovate cream (if applicable)

6.3 REPLACEMENT OF DROP-OUTS AND WITHDRAWALS

Patients dropping out from the trial will not be replaced, as the drop-out rate has already been included in the target number of recruited patients.

All withdrawals/drop-outs will be documented and the reason will be recorded in the CRF. "Terminal study status" section of the CRF will be completed for all drop-outs/withdrawals.

7 ASSESSMENT OF EFFICACY AND SAFETY

7.1 ASSESSMENT OF EFFICACY

7.1.1 PRIMARY EFFICACY PARAMETER

The primary efficacy parameter will be assessed with a comparison of the rate of AD flare during the course of the study, starting after 1 month of treatment (reporting the crisis of the month, included the one of the first visit) and during the study period, between the two groups. An AD crisis is defined as: recurrence of AD lesions quoted at least 3 according to global evaluation criteria with a minimum of 15 days from the last flare (see section 2.2)

7.1.2 SECONDARY EFFICACY PARAMETER(S)

The secondary efficacy parameters are:

- SCORAD: (see attachment 23.1)

  Comparison of SCORAD at Month 1 (considered as baseline status) and Month 3 / Month 6 / Month 9 between the 2 groups.
- Affected body area:

  Comparison of affected body surface area at Month 1 (considered as baseline status) and Month 3 / Month 6 / Month 9 between the 2 groups.
- Consumption of corticosteroid topical application during the study:

  Tubes prescribed and used will be brought back by the families at each visit. They will be accounted, weighted and compared between the 2 groups.
- Assessment by the parents/children and by the investigator

  Assessment of the global improvement of the dermatosis by answering the question "how do you judge the improvement of atopic dermatitis by the child

since the beginning of the study?» (comment jugez-vous de l'amélioration de la dermatite atopique de l'enfant depuis le début de l'étude?). The answer will be put on a scale graded with 5 items as follows: 0=null (nulle), 1=mild (peu importante), 2=moderate (modérée), 3=important (importante), 4=very important (très importante).

7.2 ASSESSMENT OF SAFETY

The following parameters will be recorded to assess the safety of the trial drug:

- General physical status at the time of examination
- Adverse events
- Serious adverse events

7.3 LABORATORY ASSESSMENTS

No laboratory assessments will be performed for the purpose of this study.

7.4 ASSESSMENT OF COMPLIANCE

Treatment compliance will be checked by means of Drug Accountability (see also section 14). The patients will be asked to return all unused study medication, including empty boxes blisters at each visit. The returned unused capsules will be counted and recorded in the CRF and on the drug accountability form (DAF) by the investigator.

The compliance is rated as good if more than 80% of the capsules have been taken, i.e. at least 24 capsules have been taken per period of 30 days, as moderate if 70% to 80% (between 21 and 23 capsules per 30 days period) have been taken, and as poor, if less than 70% of the capsules (less than 21 capsules per 30 days period) have been taken.

8 ADVERSE EVENTS

The safety of the treatment will be considered independently in each treatment group with respect to the occurrence and the characteristics of the adverse drug reactions.

Definitions 1

An adverse event (AE) is any undesirable experience occurring to a patient during the clinical trial, whether or not it is considered to be related to the trial medications. Clinically significant abnormal laboratory values are also considered as adverse events.

Adverse drug reaction (ADR) is a reaction to a drug which is noxious and unintended and which occurs at dose normally used in man for prophylaxis, diagnosis or therapy of disease or for modification of physiological function. In case of clinical trials, injuries by overdosing, abuse/dependence and interaction with other medicinal products should be considered as ADR.

Unexpected adverse event (UAE) is an experience not previously reported (in nature, severity or incidence) with the investigational product. If there is reasonable grounds for the suspicion that the UAE is causally related to the trial drug, it must be considered as ADR.

Definition 2

Serious Adverse Events (SAEs): an adverse event (AE) is defined as serious whenever the outcome is:

- Death
- Requiring inpatient hospitalisation or prolongation of existing hospitalisation
- Resulting in persistent or significant disability / incapacity
- Life-threatening
- Involving malignancy or congenital anomaly
- Medically significant

Definitions 3

- **Certain**: a clinical event, including laboratory test abnormality, occurring in a plausible time relationship to drug administration, and which cannot be explained by concurrent disease or other drugs or chemicals. The event must be plausible pharmacologically or phenomenologically, and its clinical response to withdrawal of the drug (dechallenge) should be clinically plausible. It must also be confirmed by a satisfactory rechallenge procedure.

NOTE: the decision to perform a formal rechallenge must be made by the Investigator and the Clinical Study Responsible after reviewing the patient's complete history.

- **Probable**: a clinical event, including laboratory test abnormality, with a reasonable time sequence to drug administration, unlikely to be attributed to concurrent disease or other drugs or chemicals, and which follows a clinical plausible response on withdrawal (dechallenge). Rechallenge information is not required to fulfil this definition.
- **Possible**: a clinical event, including laboratory test abnormality, with a reasonable time sequence to administration of the drug, but which could also be explained by concurrent disease or other drugs or chemicals. Information on drug withdrawal may be lacking or unclear.
- **Unlikely**: a clinical event, including laboratory test abnormality, with temporal relationship to drug administration which makes a causal relationship improbable, and in which other drugs, chemicals or underlying disease provide more plausible explanations.
- **Not assessable**: a report suggesting an adverse drug reaction, which cannot be judged because information is insufficient or contradictory, and which cannot be supplemented or verified.
- **Unrelated**: an adverse event, which is definitely not related causally to drug administration.

8.1 REPORTING AND RECORDING OF ADVERSE EVENTS

Starting from visit 2, and for all subsequent examinations during the trial, all symptoms or diseases observed by the physician or reported by the patient upon indirect questioning and which were not present at the initial examination (visit 1) must be recorded as an adverse event. Documentation should be made as to the nature, date of onset, end date, seriousness, severity, course, relation to trial medications, action(s) taken and outcome of any signs or symptoms. Clinically relevant abnormal laboratory values should be treated and documented as adverse events as well.

In any case the patient has to be followed carefully until the adverse event has resolved or at least stabilised. If there is a worsening of a medical condition that was present before starting the trial, this should be considered as an adverse event and a complete evaluation should be recorded. The relationship with the study drug to each adverse event should be determined by the investigator based on the definition detailed above.

8.2 REPORTING, RECORDING AND HANDLING OF SERIOUS ADVERSE EVENTS (SAES)

Any serious adverse event must be reported to the pharmacovigilance group of OM PHARMA immediately.

The contact person is: Dr. Gabriel Cozma
Direct line: + 41 22 783 14 71 (or + 41 22 783 11 11)
Natel: +41 79 337 76 91
Fax. + 41 22 783 14 15 (or + 41 22 783 11 22)
e-mail: gabriel.cozma@ompharma.com

All information regarding the serious adverse event must be recorded on a Serious Adverse Event (SAE) form provided by OM PHARMA (see attachment 23.7) and sent by fax to the pharmacovigilance group. The patient should be followed carefully until the condition disappears and/or the etiology has been determined and all follow-up information has been obtained and recorded on the SAE form.

In addition any SAE has to be fully documented on the AE section of the CRF.

In special cases also the Clinical Study Responsible can be contacted. He will then be responsible for transmiting the information to the pharmacovigilance group.

9 DATA MANAGEMENT AND STATISTICS

9.1 DATA MANAGEMENT

The data will be entered by an authorized person into a computer database. The data will then be re-entered by a second person and verified against the first entry. If discrepancies between the first and second entry cannot be resolved by checking the CRF, a data clarification form (DCF/query) will be generated and forwarded to the investigator for completion. Missing data will be queried as well. This allows correction of the data on the CRF working copy and the database. The DCF will be joined to the CRF.

9.2 BLIND REVIEW

Only the clinical pharmacist knows the content (drug or placebo) of the labelled boxes. Neither the patients nor the investigator know the group to which the patients have been assigned.

All data will be frozen prior to analysis and kept in a separate folder indicating the period in which the data have been produced.

At the time of the frozen procedures, data will be matched with the allocation files produced for the study; only information whether the patient has been allocated to group A or B will be available into the frozen dataset.

The allocation schedule will be masqued during the analysis: the statisticians will be blinded and only at the end of the analysis, he will know the equivalent label (drug or placebo).

9.3 SAMPLE SIZE CALCULATION

The sample size was calculated to answer the primary objective, i.e. "to prove the superiority of Imocur over placebo considering a reduction in the rate (per patient per month) of 'crisis' of atopic dermatitis of 20% and 45% in the placebo and treated group respectively ". The statistical significance is set for an α risk as usual at $p = 0.05$ for the primary variable and the power ($1-\beta$) is set to 90%.

A minimum of 84 valuable patients per treatment group will be required to show a global superiority of Imocur over placebo assuming around 20% of drop-out rate.

An exact sample size determination would require a precise estimate of the rate, which is actually unavailable.

9.4 CONFIRMATORY ANALYSIS OF EFFICACY CRITERIA

An intent-to-treat analysis will be performed, i.e. all randomized subjects, regardless of their compliance with the protocol, are to be included in the analysis (unless no trial medication has been taken at all). All statistical tests will be interpreted at the 5% significance level (2-tailed). In case of a substantial number of protocol violators, an additional per protocol analysis may be done to determine whether they influence the conclusions.

The primary efficacy parameter:

The rate of AD-'Crisis' (cumulated per patient, per month) computed at each visit up to the final visit, is the only parameter used for comparing the efficacy Imocur to that of placebo. Each patient will contribute to the rate for his specific time of permanence in the study. An AD crisis is defined as: recurrence of AD lesions quoted at least 3 according to global evaluation criteria with a minimum of 15 days since the last flare.

The hypothesis $\mathbf{H_0}$: the two treatment groups show similar levels of decrease of AD-'Crisis' rate versus the alternative $\mathbf{H_a}$: the two treatments groups show different levels of decrease of AD crisis rate, is intended to be tested by means of non parametric analysis considering also potential covariates (such as corticosteroid use).

Possible subgroups of interest in which the effect of the treatment could be investigated, should be defined. At the moment, the following variables have been selected as source of possible subgroup analysis:

- Incidence or prevalence of AD
- More or less than 3 years old.

Secondary efficacy parameters:

1. Derived from SCORAD:
    - number of children having experienced a decrease in their SCORAD of at least 40% from SCORAD at V1 and SCORAD of the more severe crisis during the study;

- Comparison of SCORAD at Month 1 (considered as baseline status) and Month 3 / Month 6 / Month 9;

2. Affected body area : body surface area is defined as affected for each of the criteria: erythema, edema/papule, oozing or crusted lesions;
   - number of children having experienced a decrease in their affected body area of at least 40% from the surface at V1 and the surface of the more severe crisis during the study;
   - comparison of affected body surface area at Month 1 (considered as baseline status) and Month 3 / Month 6 / Month 9;
3. Topical corticosteroids (as hydrocortisone-equivalents per day);
4. Assessment of the global improvement of the dermatosis by the investigator and the parents.

The two treatment groups shall be compared employing standard tests, i.e. Chi-square for nominal data and analysis of variance (ANOVA) for repeated measures, correcting for potentially confounding factors in the demographic data or randomisation blocks.

9.5 EXPLORATORY ANALYSIS OF EFFICACY CRITERIA

A validation of the primary end-point measure, the score of AD crisis, will be made analising the agreement between the crisis evaluation score at visit 1 performed by the investigator (gold standard) and the evaluation performed for the same situation specifically reported at visit 2 by the parents.

An exploratory analysis for ‘predictors of outcome’ shall be performed considering the primary end point and the changes in SCORAD as dependent variables and the demographic and baseline data, including randomisation blocks, as independent variables. These elements shall be analysed in adequate models, such as stepwise regression analysis including the potentially relevant covariates.

9.6 ANALYSIS OF SAFETY DATA

All safety data are evaluated in a descriptive manner, without adjustment according to a multiple level alpha.

Adverse events will be coded according to the WHO-ART (or MEDDRA if available) dictionary and thereafter be presented in descriptive tables according to requirements of the ICH Clinical Study Report Guideline E3. (Simple listing and listings should be subdivided according to organ class). In addition absolute frequencies will be presented with confidence intervals, analytical comparisons will be performed for all organ classes with a number of events sufficient for a quantitative interferential analysis. The experimental unit of this analysis is the patient: recurrent and also different events in the same organ class will then be counted once with the worst intensity. Serious and/or unexpected events will be separately discussed.

9.7 ANALYSED POPULATION

All patients who have had at least one dose of medication are analysed for safety. For efficacy all patients who have had at least one dose of medication and also at least one observation visit under medication are evaluated as the ´intention-to-treat´ (ITT) population. The ITT analysis is defined as the first line

analysis. A second line analysis for supportive purposes may be performed for a ´per protocol´ (PP) data set.

9.8 DATA ANALYSIS AND COMPUTER PROGRAM

Data management are planned to be carried out by using DataEase v.4 from DataEase International Inc, USA.

All the analysis will be performed using SAS® System 6.09 for OpenVMS.

9.9 INTERIM ANALYSIS

The interim safety and efficacy results of this trial will be monitored independently. There will be one formal interim analysis after 84 patients have reached the fifth visit (9 months of inclusion in the trial). The purpose of this interim analysis is to allow the termination of the trial if treatment is clearly effective. The stopping boundaries will be based on usual estimates (31) and on the consideration that this is a pilot study, using, for a conservative approach an α fixed equal to 0.01.

During this interim analysis, an evaluation of general characteristics will be performed to underline any possible selection bias.

10 ETHICAL CONSIDERATIONS AND PATIENT’S CONSENT

10.1 ETHICAL CONSIDERATIONS

The trial will be carried out in accordance with the principles stated in the Declaration of Helsinki, last revised version (52nd WMA General Assembly, Edinburgh, Scotland, October 2000 - see attachment 23.8) and in accordance with Good Clinical Practice (GCP) and applicable regulatory requirements.

10.2 ETHICS COMITTEES (EC), NATIONAL REGULATORY AUTHORITIES

According to the regulatory requirements of the country, the investigator will submit the trial documentation (protocol, CRF, informed consent, etc.) to the relevant Ethics Committee(s) (EC).

A copy of the letter of approval from the Ethics Committee, which contains a list of the names and occupations of the members of the Ethics Committee having participated at the session, as well as a list of documents reviewed, must be received by the sponsor prior to shipment of drug supplies to the investigator.

The sponsor will notify the study to the appropriate regulatory authorities as soon as the investigator has obtained the favourable opinion of the EC.

Children must not be entered into the trial until the relevant Ethics Committee has issued its opinion and the health authorities have been notified of the study as defined by local regulatory requirements.

All subsequent protocol amendments must be submitted to the Ethics Committee for approval/information.

The investigator must also inform the Ethics Committee of any serious or unexpected adverse events occurring during the trial, which are likely to affect the safety of the children or the conduct of the trial. The Ethics Committee should also be asked for its opinion if a re-evaluation of the ethical aspects of the trial appears to be called for.

The obligations of the sponsor and the investigator in conducting this trial are detailed in attachment 23.10.

10.3 INFORMED CONSENT

A child may be included into the present trial only after his/her parent/guardian has declared in a written form his/her willingness to take part in the trial. In order to be able to take this decision, the child's parent/guardian is informed previously by the investigator about the content of the planned trial as well as on the risk/benefit ratio. The information should be given both orally and in a written form. For this statement, a 'Patient's Information Sheet' (see Attachment 23.4) is available, which is in agreement in its text structure with each reference Ethics Committee's guidelines. The statement should also include indications about the insurance coverage and the resulting regulations for the delimitation of damages. The child's parent/guardian should be informed that he/she should notify the investigator of any other medical measures during the trial period and that the child cannot take part simultaneously in another trial.

The consent form (see attachment 23.5) must be dated and signed personally by the child (if appliccable) and the participant's parent/guardian (or witness/legal representative, if applicable), before any study related procedure is performed. A copy of the signed consent form as well as the patient information sheet should be given to the child's parent/guardian.

Concerning the study data, the child's parent/guardian will accept by signing the informed consent that the study data may be examined by the sponsor, the drug regulatory authorities, a mandated auditor and/or the study monitor in compliance with the statement of confidentiality.

11 INSURANCE

OM PHARMA confirms that it carries liability insurance, which protects investigators against claims for which they may become liable as a result of damages caused by OM PHARMA products used in clinical studies.

The main insurance company is:

"Zurich Assurances"

Route de Chavannes 35

CH - 1001 Lausanne

Insurance coverage is not extended to damages, which the investigators or third parties may suffer by reason of acts of commission or omission on accepted common medical practices (lege artis procedures).

12 QUALITY ASSURANCE / AUDIT

Quality assurance audits of this trial may be conducted by the sponsor or a mandated auditor. GCP audits can also be performed by the authorities. The quality assurance auditor should have access to all medical records, the investigator's trial related files and correspondence, and the informed consent documentation that is relevant to this clinical trial.

13 PATIENT DOCUMENTATION AND MONITORING

13.1 CASE REPORT FORM (CRF)

CRFs for individual subjects will be provided by the sponsor. Each page is printed on NCR paper. The bottom copy remains with the investigator as a permanent record. The original and the first copy will be returned to the sponsor.

Children are identified on the CRF only by appropriate coded identification (e.g. subject number) and subject initials. CRFs are used to record clinical trial data and are an integral part of the trial and subsequent reports. The entries, therefore, must be legible and complete. The CRF can be completed by the investigator/authorised persons (mentioned in the centre study personnel identification form). Errors should be lined out but not obliterated and the correction inserted, initialled and dated. Units of measurement other than those requested on the CRF must always be clearly indicated. All data fields must be completed. Any additional comments should also be initialled and dated. The declaration ensuring accuracy of all data recorded in the CRF must be signed by the investigator/authorised person on the last page of the CRF.

CRFs must be completed after each visit to reflect subject status during the course of the trial. The monitor reviews and collects the completed CRF pages and forwards them to the data management.

Documented medical histories and narrative statements relative to the subject's progress during the trial will be maintained by the investigator. These records should also include patient’s diary cards, which must be kept on file with the individual patient's case report forms.

Examinations especially performed for trial purposes, as precised in this protocol are physical examination, including dermatological diagnosis. These data are reported directly on the CRF and are considered as being source data.

13.2 MONITORING

The sponsor assigns monitors for on-site monitoring. These monitors are working directly for the sponsor company. Monitors have to work according to Standard Operating Procedures (SOPs) of the sponsor. Monitoring visits will be performed between initiation visit and close out visit at regular intervals.

The monitor ensures the completeness, correctness, consistency with source data and the legibility by reviewing the Case Report Forms (CRF) entries according to SOPs. For this tasks the monitor must be allowed to have access to the source data.

CRFs progress notes and copies of medical test results must be available at all times for inspection by the clinical trial monitor and health authorities. The monitor will review all CRFs and written informed consents.

14 DRUG ACCOUNTABILITY

The investigator must maintain accurate and adequate records including dates, batch number, quantities received, individual usage, etc. He/she must also return to the sponsor unused supplies giving an exact amount of usage in the trial whether completed or terminated. At the time of return to the sponsor, the investigator must verify that all unused or partially used drug supplies have been returned by the clinical trial subjects and that no remaining supplies are in the investigator's possession.

15 ADMINISTRATIVE ISSUES

15.1 RULES FOR AMENDING THE PROTOCOL

Any change to the protocol, once the final version has been issued, has to be detailed in a protocol amendment. All protocol amendments must be numbered, dated and signed at least by the investigator and the sponsor.

All protocol amendments must be reviewed by the Ethics Committee with a written approval and submitted to the local authorities, if required. Purely administrative amendments can be sent to the ECs for information only. Documentation of this review must be provided to the sponsor.

15.2 DISCONTINUATION OF THE TRIAL BY THE SPONSOR

The sponsor reserves the right to discontinue this trial at any time for insufficient patient recruitment and any other reasons. The investigator will be reimbursed for reasonable expenses incurred if it is necessary to terminate the trial.

15.3 ARCHIVING

The investigator must keep the trial specific patient documentation for at least 15 years after termination of the trial. The investigator should take measures to prevent accidental or premature destruction of the study documents as long as they have to be maintained according to the applicable regulatory requirements and at least until the sponsor has informed him/her that they no longer need to be retained.

16 STATEMENT OF CONFIDENTIALITY

Individual patient medical information obtained as a result of this study is considered confidential and disclosure to third parties is prohibited. Subject confidentiality will be further ensured by utilizing subject identification code numbers to correspond to patient data in the computer files.

Data generated as a result of this trial are to be made available for inspection/audit on request of the Sponsor, the Ethics Committee and/or the regulatory health authorities.

17 SECRECY AGREEMENT

The investigator commits himself to keep secret from third parties any confidential information obtained from and concerning OM PHARMA or this company's products, which in connection with the present contractual relationship are made available or disclosed, respectively, and to use this knowledge only as agreed upon.

This commitment is valid independent of the existence and the duration of the present actual relationship, but only so far and so long as OM PHARMA is reasonably and justly interested in the investigator's maintaining this secrecy undertaking.

18 REPORTING AND PUBLICATION

18.1 REPORTING

A final report (integrated medical and statistical study report) will be prepared by the sponsor according to ICH E3. This task can also be delegated to a CRO. The final study report will be regarded as confidential and may be submitted to regulatory authorities world-wide for registration purposes.

18.2 PUBLICATION

OM PHARMA has no objections to the investigator's publishing the results of this study. As a general principle such a publication should be based on the integrated medical and statistical study report, and therefore the publication should be submitted only after finalisation of the study report. The investigator will submit a copy of the manuscript to OM PHARMA for review, at least 30 days before submitting a manuscript to the publisher. Such a review is necessary to prevent premature disclosure of trade secrets or otherwise patent-protected material, and has no restrictive intentions concerning facts or opinions of the investigator.

It is intended that this study will be published as a whole by the principal investigator while mentioning the co-investigators as co-authors.

19 FINANCIALS

OM PHARMA’s financial obligations are outlined in a separate agreement. If OM PHARMA discontinues the study prematurely for administrative reasons, reasonable expenses will be remitted.

20 CONTINUATION SUPPLY

No continuation supplies will be available to children who complete the trial.

21 SCIENTIFIC AGREEMENT / SIGNATURES

21.1 AGREEMENT

The protocol should be signed and dated at least by the Study Co-ordinator, the Sponsor, represented by the Head of Medical and Regulatory Affairs, the Clinical Study Responsible, the Biostatistician and the Contract Research Organization.

21.2 SIGNATURES

By signing the document, the investigator confirms that he/she understood the investigator brochure including the risks and drug related adverse events of the drug(s). He/she agrees to conduct the study as outlined in the above protocol, which contains all details necessary for carrying out the study. The investigation will be completed within the agreed time scheduled.

SIGNATURES PAGE

STUDY CO-ORDINATORS

Professor Yves de Prost

Signature: ________________________ Date: ________________________

Professor Christine Bodener

Signature: ________________________ Date: ________________________

OM PHARMA

Head of Medical and Regulatory Affairs

Dr. G. Cozma, MD

Signature: ________________________ Date: ________________________

Clinical Study Responsible

Stephane Deshormieres

Signature: ________________________ Date: ________________________

BIOSTATISTICIAN

Liliane Marie Chatenoud

Signature: ________________________ Date: ________________________

Pharma Part (CRO)

Urs Billeter

Signature: ____________________ Date: ____________________

22 REFERENCES

1. Cooper, K.D, 1994. Atopic dermatitis: recent trends in pathogenesis and therapy. *J. Invest. Dermatol. 102:128.*
2. Horan, R.F, Scneider L.C, Sheffer A.L. 1992. Allergic skin disorders and mastocytosis. *JAMA 268:2858.*
3. Ohmen, J.D, Hanifin J.M, Nickoloff B.J, Rea T.H, Wyzykowski R, Kim J, Jullien D, Mchugh T, Nassif A.S, Chan S.C, et al. 1995. Overexpression of IL-10 in atopic dermatitis: contrasting cytokine patterns with delayed-type hypersensitivity reactions. *J.Immunol. 154:1956.*
4. Kay, A.B, Ying S, Varney V, Gaga M, Durham S.R, Moqbel R, Wardlaw A.J, Hamid Q. 1991. Messenger RNA expression of the cytokine gene cluster, interleukine 3(IL-3), IL-4, IL-5 and granulocyte/macrophage colony-stimulating factor, in allergen-induced late-phase cutaneous reactions in atopic subjects. *J. Exp. Med. 173:775.*
5. Hanifin JM, Rajka G. 1980. Diagnostic features of atopic dermatitis. *Acta Derm Venereol; suppl 92:44-47.*
6. Williams HC, Burney PGJ, Hay RJ, et al. 1994. The UK working party's diagnostic criteria for atopic dermatitis, I: derivation of a minimum set of discriminators for atopic dermatitis. *Br J Dermatol; 131:383-396.*
7. Wolkerstorfer A, de Waard van der Spek FB, Glazenburg EJ, Mulder PG, Oranje AP. 1999. Scoring the severity of atopic dermatitis: three ite severity score as a rough system for daily practice and as pre-screening tool for studies. *Acta Derm Venereol; 79:356-9.*
8. Wybran J, Libin M, Schandene L. 1989. Activation of natural killer cells and cytokine production in man by bacterial extracts. *Immunopharmacol Immunotoxicol; 11: 17-32.*
9. Mauel J, Van Pham T, Kreis B, Bauer J. 1989. Stimulation by a bacterial extract (Broncho-Vaxom) of the metabolic and functional activities of murine macrophages. *Int J Immunopharmacol; 11: 637-645.*
10. Fontanges R, Bottex C, Cristau B, Burckhart M F. 1990. Influence of a bacterial extract on antigen presentation and protection against experimental infections. *Lung; 168: 716-719.*
11. Duchow J, Marchant A, Delville JP, Schandene L, Goldman M. 1992. Upregulation of adhesion molecules induced by Broncho-Vaxom on phagocytic cells. *Int J Immunopharmacol; 14: 761-766.*
12. Puigdollers JM, Serna GR, Hernandez del Rey I, Barruffet MT, Torroella JJ. 1980. Immunoglobulin production in man stimulated by an orally administered bacterial lysate. *Respiration; 40: 142-149.*
13. Emmerich B, Emslander HP, Milatovic D, Hallek M, Pachmann K. 1990. Effects of a bacterial extract on local immunity of the lung in patients with chronic bronchitis. *Lung; 168: 726-731.*
14. Lusuardi M, Capelli A, Carli S, Spada EL, Spinazzi A, Donner CF. 1993. Local airways immune modifications induced by oral bacterial extracts in chronic bronchitis. *Chest; 103: 1783-1791.*

15. Martin du Pan RE, Martin du Pan RC. 1982. Clinical study concerning the prevention of infections of the upper respiratory tract of preschool children. *Schweiz Rundsch Med Prax; 71: 1385-1389.*
16. Ahrens J. 1984. Klinische Wirksamkeit eines oralen Immuntherapeutikums bei Kindern mit rezidivierenden Atemwegsinfektionen. *Therapiewoche; 34: 3469-3475.*
17. Zagar S, Lofler Badzek D. 1988. Broncho-Vaxom in children with rhinosinusitis: a double-blind clinical trial. *ORL; 50: 397-404.*
18. Maestroni GJ, Losa GA. 1984. Clinical and immunobiological effects of an orally administered bacterial extract. *Int J Immunopharmacol; 6: 111-117.*
19. Martinez F.D, Holt P.G. 1999. Role of microbial burden in aetiology of allergy and asthma. *Lancet; 354 Suppl 2:SII12-5.*
20. Holt P.G, Macaubas C, Stumbles P.A,Sly P.D. 1999. The role of allergy in the development of asthma. *Nature; 402(6760 Suppl): B12-7.*
21. Wahn U, Von Mutius E. 2001. Childhood risk factors for atopy and the importance of early intervention. *J.Allergy Clin Immunol; 107(4):567-74.*
22. Von Ehrenstein O.S, Von Mutius E, Illi S and al. 2000. Reduced risk of hay fever and asthma among children of farmers. *Clin Exp Allergy; 30(2):187-93.*
23. Bowman L.M, Holt P.G. 2001. Selective enhancement of systemic Th1 immunity in immunologically immature rats with an orally administered bacterial extract. *Infection and Immunity, 69 (6), 3719-3727.*
24. Bessler W.G. OM-85 activates macrophages and modulates cytokines and IgE response in orally immunized mice. May 29, 2001. *Experimental report*.
25. Podleski W.K. 1985. Immunomodulation of allergic autocytotoxicity in bronchial asthma by a bacterial lysate – Broncho-Vaxom. *Int. J. Immunopharmac.,7, 713.*
26. Fontanges R, Bottex C, Murckart M.F, Gerasimo P, Mathieu J. Broncho-Vaxom's mechanism of action. *7th Cong. Eur. Soc. Pneumology (SEP), 5-9 Sept. 1988, Budapest. Hungary.*
27. Keller R, Hinz G. 1984. Effect of an oral polyvalent bacterial lysate (Broncho-Vaxom®) in chronic bronchitis. *Prax. Klin. Pneumol., 38, 225-228.*
28. Emmerich B et al. Immunomodulation with bacterial extracts: Effects on gamma interferon, macrophages, T-lymphocytes and S-IgA in bronchoalveolar lavage fluid of patients with chronic bronchitis. *7th Cong. Eur. Soc. Pneumology (SEP), 5-9 Sept. 1988, Budapest. Hungary.*
29. Djuric D, Mihailovic-Vucinic V, stojcic V. 1989. Effect of Broncho-Vaxom on clinical and immunological parameters in patients with chronic obstructive bronchitis. A double-blind, placebo-controlled study. *Int. J. Immunother., V, 139-143.*
30. Pocock S. J. 1977. Group sequential methods in the design and analysis of clinical trials. *Biometrika, 64, 191-199.*
31. Consensus Report of the European Task Force on Atopic Dermatitis. 1993. Severity Scoring of Atopic Dermatitis: The SCORAD Index. *Dermatology; 186:23-31.*

23 ATTACHMENTS TO THE PROTOCOL

23.1 SCORAD INDEX

SCORAD index

Analysis of surface involvement

1. During the clinical examination, draw the involved areas on the evaluation sheet.
2. Then calculate the proportion of involved surface area segment by segment.
3. Total the results for all segments.

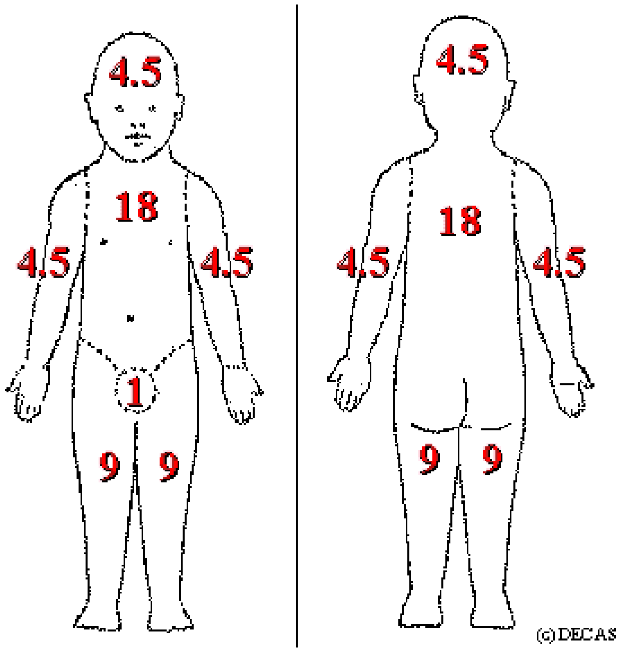

A = Area involved: |___|___|___|/100

Grading of intensity

1. Choose the representative area

   In patients with localised lesions, analysis will concern the area which was the reason for the visit.
   In patients with areas varying in severity, a representative area of mean involvement is chosen.
   Dryness should be evaluated in a remote area of non-inflammatory skin.

2. Identify the elementary lesions

Criteria	Intensity
Erythema	|___|
Edema/papulation	|___|
Oozing/crust	|___|
Excoriation	|___|
Lichenification	|___|
Dryness*	|___|

Means of calculation

Intensity items (average representative area)

0 = absence ; 1 = mild ; 2 = moderate ; 3 = severe

*Dryness is evaluated on uninvolved areas

B = Intensity : |___|___|/18

Subjective signs

The two most representative items concerning the quality of life of patients (during the 3 previous days) are :

- Pruritus
- Insomnia

The patient and/or one of his relatives expresses the intensity of pruritus or insomnia using a visual analogue scale graded between 0 and 10.

Pruritus: |___|___|/10

Insomnia: |___|___|/10

C = Subjective symptoms : pruritus + sleep loss: |___|___|/20

SCORE TOTAL SCORAD: A/5 + 7B/2 + C: |___|___|___|/103

23.2 THE CRITERIA OF HANIFIN AND RAJKA

THE CRITERIA OF HANIFIN AND RAJKA

Guidelines for the diagnosis of atopic dermatitis

Must have 3 or more basic features:

- Pruritus
- Typical morphology and distribution:
  - ° Flexural lichenification or linearity in adults.
  - ° Facial and extensor involvement in infants and children
- Chronic or chronically-relapsing dermatitis
- Personal or family history of atopy (asthma, allergic rhinitis, atopic dermatitis)

Plus 3 or more minor features:

- Xerosis
- Ichthyosis/palmar hyperlinearity/keratosis pilaris
- Immediate (type I) skin test reactivity
- Elevated serum IgE
- Early age of onset
- Tendency toward cutaneous infections (esp. *Staph. Aureus* and *Herpes simplex*)/ impaired cell-mediated immunity
- Tendency toward non-specific hand or foot dermatitis
- Nipple eczema
- Cheilitis
- Recurrent conjunctivitis
- Dennie-Morgan infraorbital fold
- Keratoconus
- Anterior subcapsular cataracts
- Orbital darkening
- Facial pallor/ facial erythema
- Pityriasis alba
- Anterior neck folds
- Itch when sweating
- Intolerance to wool and lipid solvents
- Perifollicular accentuation
- Food intolerance
- Course influenced by environmental/ emotional factors
- White dermographism/ delayed blanch

THE CRITERIA OF WILLIAMS ET AL

THE CRITERIA OF WILLIAMS ET AL

AD diagnosis requires:

- An itchy condition (or parental report of scratching or rubbing in a child)

Plus 3 or more of the following

- History of involvement of the skin creases such as folds of elbows, behind the knees, fronts of ankles or around the neck (including cheeks in children under 10).
- A personal history of asthma or hay fever (or history of atopic disease in a first-degree relative in children under 4).
- A history of a general dry skin in the last year.
- Visible flexural eczema (or eczema involving the cheeks/forehead and outer limbs in children under 4).
- Onset under the age of 2 (not used if child is under 4).

23.3 WRITTEN PATIENT INFORMATION SHEET

INFORMATION DESTINEE AU PATIENT

Efficacité et tolérance d'Imocur (OM-85 BV) chez des enfants souffrant de dermatite atopique. Etude multicentrique, randomisée, en double aveugle contre placebo.

Madame, Mademoiselle, Monsieur,

Votre enfant souffre de dermatite atopique qui est une dermatose inflammatoire chronique qui débute chez l'enfant dans les premiers mois de vie, généralement vers 3 mois, parfois plus précocement. L'aspect des lésions est variable selon la gravité de la dermatite atopique et le moment de l'examen (poussée ou rémission).

Il existe différents stades de sévérité d'une poussée à l'autre chez un même enfant, et d'un enfant à l'autre, au cours de l'évolution de cette dermatose inflammatoire. Des critères de sévérité peuvent être définis précisément grâce à différentes méthodes durant l'examen clinique, qui tiennent compte de l'étendue de la poussée d'eczéma, de la sévérité des lésions et de l'importance des démangeaisons.

A l'heure actuelle, il n'existe aucun traitement définitif de cette dermatose inflammatoire chronique débutant dans la petite enfance, pouvant se poursuivre jusqu'à l'âge adulte. Le traitement de référence le plus classique est un traitement par dermocorticoïdes, prescrits si la poussée inflammatoire le justifie. Les dermocorticoïdes sont utilisés pour traiter les poussées de la maladie. Ils ne sont que symptomatiques, n'évitent pas les réapparitions de l'affection, et leur utilisation au long cours a des effets secondaires bien connus qui peuvent poser un problème important chez l'enfant, et en particulier le nourrisson.

Imocur est un traitement déjà utilisé chez l'enfant, et même chez le nourrisson, à partir de 6 mois, en prévention d'infections rhino-pharyngées à répétition, avec une très bonne tolérance clinique. Chez l'homme et l'animal, il a été montré qu'Imocur, constitué de 8 fractions bactériennes non agressives, pouvait moduler positivement le système immunitaire (défenses naturelles de l'organisme).

Ces données permettent de penser qu'un traitement avec Imocur, en permettant une mise en contact précoce avec des dérivés bactériens inoffensifs, d'enfants atteints de dermatite atopique, pourrait en modulant les défenses naturelles de ces enfants, jouer un rôle actif dans la prise en charge thérapeutique de leur dermatite atopique.

Description de l'étude

Le but de cette étude est d'évaluer l'efficacité et la tolérance d'Imocur, en comparaison avec une substance sans principe actif, chez des enfants souffrant de dermatite atopique. Cette efficacité sera déterminée par la diminution du nombre de poussées et par une diminution de l'intensité de ces crises (utilisation de l'échelle SCORAD).

L'étude est conçue de telle façon que la moitié des participants à l'étude reçoit une capsule sans principe actif (placebo). Le tirage au sort repose sur le hasard (randomisation) et ni l'enfant, ni le médecin ne connaîtront l'attribution des produits (ce qu'on appelle le double aveugle). Ce procédé selon lequel seule une moitié des enfants reçoit le médicament et l'autre moitié un placebo est nécessaire pour obtenir une image objective de l'efficacité du médicament.

Même si votre enfant fait partie des patients recevant la préparation sans principe actif, il profitera d'une surveillance médicale étroite telle que prévue par l'étude.

Sélection des participants a l'étude

Votre enfant a été retenu pour participer à cette étude car il présente une dermatite atopique caractérisée par un SCORAD compris entre 25 et 70 et affectant entre 15 et 70% de sa surface corporelle. Seuls des enfants âgés entre 6 mois et 7 ans ont été inclus dans cette étude.

Environ 210 enfants participent à cette étude répartis dans 8 centres en France.

Déroulement de l'étude

L'étude dure en tout 12 mois.

Le traitement sera administré sous forme de gélules, avec une seule gélule par jour pendant 9 mois. Il sera possible d'ouvrir la gélule de manière à diluer son contenu dans un liquide (lait, jus de fruit…) pour le confort de votre enfant.

Pendant toute la durée de l'étude, la dermatite atopique de l'enfant sera traitée avec un traitement conventionnel à base de corticoïdes. De manière à harmoniser ce traitement, Dexeryl (1 application par jour pendant toute l'étude) et Flixovate crème (1 application par jour pendant 8 jours puis 1 application tous les 2 jours pendant 8 jours et enfin 1 application tous les 3 jours pendant 8 jours) seront utilisés. En ce qui concerne Flixovate crème, une fois la période de traitement terminée, vous ne devrez en aucun cas réutiliser ce produit sans ordonnance de la part du médecin investigateur (critère important de l'étude).

L'enfant sera suivi par le médecin investigateur pendant toute la durée de l'étude. Vous devrez le présenter à 6 visites médicales qui auront lieu à 0, 1, 3, 6, 9 et 12 mois afin de recevoir le médicament et de vérifier la bonne observance et la bonne tolérance du traitement. Au cours de la première visite, le médecin investigateur fera un examen clinique complet de l'enfant et vous questionnera sur ses antécédents médicaux pour s'assurer qu'il remplisse les critères d'entrée pour cette étude. Il vous donnera alors le traitement nécessaire jusqu'à la visite suivante plus une boîte de réserve qui servira en cas de retard des visites suivantes, ainsi qu'un carnet de jour qu'il vous faudra remplir quotidiennement.

A la visite suivante (après 1 mois), le médecin reprendra les médicaments non utilisés (ne pas oublier de ramener également la boîte de réserve) ainsi que les emballages vides et vous redonnera la durée de traitement nécessaire. Il vous sera demandé de remplir des questionnaires concernant son état de santé durant la période écoulée et vous serez interrogé(e) sur la survenue d'éventuels effets indésirables. La visite consistera en un examen clinique de façon à apprécier l'évolution de la dermatite atopique.
Il n'y a aucun examen biologique (prise de sang ou d'urine) et la plupart des examens médicaux sont ceux habituellement faits dans le cas de la maladie. Ces visites seront répétées aux mois 3, 6 et 9.

Le traitement durant 9 mois, une dernière visite est prévue au 12ème mois de façon à suivre l'état de santé de votre enfant après l'arrêt du traitement.

Si votre enfant fait une poussée de dermatite atopique entre 2 visites, une visite chez le médecin investigateur sera nécessaire et cette visite sera considérée comme une visite intermédiaire. Dans ce cas, le traitement avec Flixovate crème sera alors réévalué.

Risques éventuels

Imocur n'a jamais été utilisé chez l'enfant dans le traitement de la dermatite atopique. Dans ses autres indications, Imocur est généralement bien toléré mais comme pour tout autre médicament, il est possible que des effets secondaires surviennent. Ceux-ci sont observés lors d'études cliniques chez 3% à 4% des malades traités. Ces effets secondaires ont toujours été sans gravité et consistent en des gastralgies, des nausées et de la diarrhée. Des éruptions cutanées peuvent également survenir ainsi que des maux de tête et plus rarement de la fatigue et des crises de toux. La plupart du temps, ces effets indésirables disparaissent à l'arrêt du traitement. Votre enfant bénéficiera de soins immédiats et appropriés si des problèmes survenaient.

Bénéfices attendus

Il est attendu du traitement par Imocur une amélioration de l'état de votre enfant. Les études cliniques sont nécessaires pour accroître les connaissances sur l'efficacité et la tolérance des médicaments.

Nouvelles découvertes

Le médecin vous informera au cours de l'étude, de toute nouvelle découverte qui pourrait influencer les bénéfices ou la sécurité de l'étude ainsi que votre déclaration de consentement.

Confidentialité

En application de la loi Informatique et Libertés du 6 janvier 1978, modifiée par la loi n° 94-548 du 1er juillet 94, les données enregistrées à l'occasion de cette étude pourront faire l'objet d'un traitement informatisé par le promoteur. Vous pourrez exercer un droit d'accès (articles 34 et 40) et de rectification (article 36) ouvert par les textes susvisés à tout moment auprès du médecin investigateur qui suivra votre enfant durant cette étude.

Protection / Assurance

Durant la participation de votre enfant à l'étude, il bénéficie d'une assurance spéciale couvrant les frais du traitement médical pour tous les problèmes de santé qui pourraient survenir au cours de l'étude. Votre médecin entreprendra alors toutes les démarches nécessaires.

Participation

La participation à cette étude est volontaire. Vous pouvez refuser de faire participer votre enfant à cette étude ou vous pouvez à tout moment le sortir de l'étude sans conséquence et sans avoir besoin de vous justifier. Votre médecin peut également décider de faire sortir votre enfant de l'étude à n'importe quel moment s'il pense qu'il est de son intérêt de ne pas continuer. Dans tous les cas, votre enfant subira un examen médical final pour sa sécurité.

Il est important que votre enfant prenne convenablement son traitement. Pour ne pas compromettre la santé de votre enfant, il ne devra prendre un autre traitement médical pendant la duré de l'étude qu'avec l'accord du médecin investigateur, à l'exception bien sûr des cas d'urgences.

Conformément à la loi, si vous acceptez de participer à l'étude, votre médecin vous demandera de signer et dater le formulaire de consentement ci-joint.

L'équipe médicale est à votre disposition pour répondre à toutes les questions que peut soulever la participation de votre enfant à cette étude.

Alternative thérapeutique

Si vous ne souhaitez pas que votre enfant participe à cette étude, le médecin investigateur discutera avec vous des autres traitements possibles pour sa dermatite atopique.

Avis du Comité Consultatif de Protection des Personnes qui se prêtent à la recherche Biomédicale (CCPPRB)

Le CCPPRB de l'hôpital Paris-Necker a été consulté et a donné un avis favorable sur cette étude.

Personne à contacter

En cas d'incertitude, de besoin, d'événements inattendus ou indésirables survenant pendant ou après l'étude, vous pouvez à tout moment vous adresser à:

Nom:………………………………………….
Adresse:……………………………………
Numéro de tel:…………………………………

Date: Signature des parents / représentant légal:

Eventuellement : Signature de l'enfant :

23.4 SAMPLE OF INFORMED CONSENT FORM

Participation à une étude clinique: déclaration écrite de consentement

- ***Veuillez lire attentivement ce formulaire.***
- ***Pour toute explication ou toute information complémentaire, n'hésitez pas à poser des questions.***

**Numéro de l'étude: BV-2002/1**
**Intitulé de l'étude: «**Efficacité et tolérance d'Imocur (OM-85 BV) chez des enfants souffrant de dermatite atopique. Etude multicentrique, randomisée, en double aveugle contre placebo.**»**
**Lieu de déroulement de l'étude:**
**Investigateur**<br>Nom et prénom:
**Patient**<br>Nom et prénom:
**Parents ou Représentant légal du patient**<br>Nom et prénom:<br>Nom et prénom:
Lien de parenté éventuel avec le représentant légal:

- Le médecin signataire m'a informé(e) oralement et par écrit des buts de l'étude portant sur l'efficacité et la tolérance d'Imocur, de son déroulement, des effets attendus, des avantages et inconvénients possibles ainsi que des risques éventuels.
- J'ai lu et compris le dossier d'information du patient pour l'étude susnommée. J'ai reçu des réponses satisfaisantes aux questions concernant la participation de mon enfant à cette étude. Je peux garder le formulaire d'information destiné au patient et je reçois une copie de ma déclaration écrite de consentement.
- J'ai eu suffisamment de temps pour prendre ma décision.
- Je sais qu'une assurance couvre les éventuels problèmes de santé qui pourraient survenir au cours de l'étude.
- J'accepte le fait que les spécialistes responsables travaillant pour le promoteur de l'étude, les représentants des autorités et des commissions d'éthique aient un droit de regard sur les données originales concernant mon enfant pour procéder à des vérifications, ces informations restant toutefois strictement confidentielles.
- Mon enfant participe volontairement à cette étude. Je peux à tout moment retirer mon accord de participation à cette étude sans avoir à donner de raisons. Dans ce cas, mon enfant subira un examen médical final pour sa propre sécurité. Aucun inconvénient pour son suivi médical ultérieur ne doit découler de cette décision.
- Je sais que les exigences et restrictions mentionnées dans le dossier d'information du patient doivent être respectées durant l'étude. Dans l'intérêt de sa santé, l'investigateur peut à tout moment décider d'exclure mon enfant de l'étude. C'est pourquoi j'informerai l'investigateur d'un éventuel traitement simultané chez un autre médecin ainsi que de la prise de médicaments (prescrits par le médecin ou achetés de ma propre initiative).

Lieu, date et signature des parents ou du représentant légal	
Eventuellement: signature de l'enfant	
Lieu, date et signature de l'investigateur	

23.5 INSURANCE CERTIFICATE

ZURICH

Zurich International France
Service clients, RC internationale
Nom du signataire : Héléna Gérard
Tél : 01-44-15-52-92
Fax : 01-44-15-07-42

ATTESTATION D'ASSURANCE RESPONSABILITE CIVILE PROMOTEUR DE RECHERCHE BIOMEDICALE

Nous soussignés, **ZURICH INTERNATIONAL** (France), dont le Siège Social est sis à Paris (17ème), 19 rue Guillaume Tell, attestons que :

OM Pharma
22 rue du Bois-du-Lan
1217 Meyrin 2 / Genève

est assuré par un contrat de Responsabilité Civile souscrit auprès de notre Compagnie sous le n° **07.400.486** G conformément aux dispositions de la Loi N 88 -1138 du 20 Décembre 1988, modifiée par la loi 90-86 du 23 Janvier 1990, le Décret 91-440 du 14 Mai 1991 et la Loi 94-630 du 25 Juillet 1994 et le décret 97-888 du 1er octobre 1997.

Objet de la recherche assurée : Multicentric, randomised, double blind, placebo-controlled study of the efficacy and safety of Imocur ® (OM-85 BV) in children suffering from atopic dermatitis

Protocole : BV-2002/1

Phase concernée : III

Date de début de recherche : 1er janvier 2003

Date de fin de recherche : 30 juin 2005

Nombre de sujets prévus : 256

La recherche étant menée conformément aux dispositions légales et réglementaires la concernant, la garantie sera acquise dans les termes du Décret 91-440 dès l'obtention de l'avis favorable du Comité Consultatif, et ce pendant toute la durée effective de la recherche.

La présente attestation est délivrée pour être remise au Comité Consultatif de protection des personnes.

Elle ne saurait engager l'assureur au-delà des clauses et conditions du contrat auquel elle se réfère.

Fait à Paris, le 8 octobre 2002
Pour servir et valoir ce que de droit
Pour la Compagnie,

Zurich International (France)
19, rue Guillaume Tell
75808 PARIS Cedex 17

23.6 SERIOUS ADVERSE EVENT REPORT FORM (FOR SAE REPORTING)

SERIOUS ADVERSE EVENT REPORT FORM

**Study reference**:	**Site number** (if applicable)	**Patient's initials**	**Randomisation number**
	|___|___|___|	|___|___|___|	|___|___|___|___|

Outcome/consequences of adverse event (check one):

☐ Recovered without sequelae ☐ Recovered with sequelae* ☐ Not yet recovered
☐ Fatal ☐ Unknown/lost to follow-up

*Sequelae: ______

Date of death: |___|___|___|___|___|___| (Day Month Year) **Cause of death**: ______

Autopsy performed: ☐ Yes ☐ No

Relevant concomitant medications: ☐ Yes ☐ None

Identify concomitant medications (list medications taken at the time of the event and 14 days prior to the event). Do not include medications used to treat the adverse event:

Medication	Daily dose	Start date	Stop date or ongoing	
		|___|___|___|___|___|___| Day Month Year	|___|___|___|___|___|___| Day Month Year	☐
		|___|___|___|___|___|___| Day Month Year	|___|___|___|___|___|___| Day Month Year	☐
		|___|___|___|___|___|___| Day Month Year	|___|___|___|___|___|___| Day Month Year	☐
		|___|___|___|___|___|___| Day Month Year	|___|___|___|___|___|___| Day Month Year	☐
		|___|___|___|___|___|___| Day Month Year	|___|___|___|___|___|___| Day Month Year	☐

Corrective therapy (describe any medical interventions, medications and/or surgical treatments used **to treat the event**):

______

______

______

Relevant medical history and concomitant diseases: ☐ Yes ☐ None

Condition	Start date	Stop date or ongoing	
	|___|___|___|___|___|___| Day Month Year	|___|___|___|___|___|___| Day Month Year	☐
	|___|___|___|___|___|___| Day Month Year	|___|___|___|___|___|___| Day Month Year	☐
	|___|___|___|___|___|___| Day Month Year	|___|___|___|___|___|___| Day Month Year	☐
	|___|___|___|___|___|___| Day Month Year	|___|___|___|___|___|___| Day Month Year	☐
	|___|___|___|___|___|___| Day Month Year	|___|___|___|___|___|___| Day Month Year	☐

Enclosures: Hospital report ☐ Autopsy report ☐ Relevant lab. tests ☐ Additional information ☐

Investigator's signature and stamp:

______ **Date signed:** |___|___|___|___|___|___| (Day Month Year)

SERIOUS ADVERSE EVENT REPORT FORM (To use for immediate report)

**Study reference:**	**Site number** (if applicable)	**Patient's initials**	**Randomisation number**
Type of report<br>☐ **Initial** ☐ **Follow-up**	|___|___|___|	|___|___|___|	|___|___|___|___|

Sex: ☐ Male ☐ Female **Date of birth:** |___|___|___|___|___|___| **Weight (kg):** |___|___|___|

Day Month Year

Height (cm): |___|___|___|

Primary diagnosis of the SAE if known (otherwise list symptoms in order of appearance):

Onset date: |___|___|___|___|___|___| **End date:** |___|___|___|___|___|___| **or ongoing:** ☐

Day Month Year Day Month Year

Serious criteria (check all that apply):

☐ Death ☐ Life-threatening

☐ Required or prolonged hospitalisation; if yes from |___|___|___|___|___|___| to |___|___|___|___|___|___|

Day Month Year Day Month Year

☐ Persistent or significant disability/incapacity ☐ Malignancy/congenital anomaly ☐ Medically significant

Details of the event:

Study drug information:

Study drug name: ______________________ **Daily dose:** _______________ **Route:** _________________

Batch number: _________________ **Expiry date:** ___________________

Start date: |___|___|___|___|___|___| **Stop date:** |___|___|___|___|___|___| **or ongoing:** ☐

Day Month Year Day Month Year

Action taken regarding study drug (check one):

☐ None ☐ Reduced ☐ Permanent discontinuation ☐ Interrupted*

*If "Interrupted", from: |___|___|___|___|___|___| to: |___|___|___|___|___|___|

Day Month Year Day Month Year

Did event abate after drug discontinuation ? ☐ Yes ☐ No ☐ Not applicable

Did event reappear after reintroduction ? ☐ Yes ☐ No ☐ Not applicable

Was the blind broken ? ☐ Yes ☐ No

Relationship to study drug:

In your medical judgement, can you assess the causality between the adverse event and the study drug ?

☐ Unrelated ☐ Not assessable ☐ Unlikely ☐ Possible ☐ Probable ☐ Certain

Comments on causality:

SERIOUS ADVERSE EVENT REPORT FORM

**Study reference:**	**Site number** (if applicable)	**Patient's initials**	**Randomisation number**
	|___|___|___|	|___|___|___|	|___|___|___|___|

Additional information

Investigator's signature and stamp:

____

Date signed: \|___\|___\|___\|___\|___\|___\|
Day Month Year

23.7 DECLARATION OF HELSINKI

WORLD MEDICAL ASSOCIATION DECLARATION OF HELSINKI

Ethical Principles for Medical Research Involving Human Subjects

Adopted by the 18th WMA General Assembly
Helsinki, Finland, June 1964
and amended by the
29th WMA General Assembly, Tokyo, Japan, October 1975
35th WMA General Assembly, Venice, Italy, October 1983
41st WMA General Assembly, Hong Kong, September 1989
48th WMA General Assembly, Somerset West, Republic of South Africa, October 1996
and the
52nd WMA General Assembly, Edinburgh, Scotland, October 2000

A. INTRODUCTION

1. The World Medical Association has developed the Declaration of Helsinki as a statement of ethical principles to provide guidance to physicians and other participants in medical research involving human subjects. Medical research involving human subjects includes research on identifiable human material or identifiable data.
2. It is the duty of the physician to promote and safeguard the health of the people. The physician's knowledge and conscience are dedicated to the fulfillment of this duty.
3. The Declaration of Geneva of the World Medical Association binds the physician with the words, "The health of my patient will be my first consideration," and the International Code of Medical Ethics declares that, "A physician shall act only in the patient's interest when providing medical care which might have the effect of weakening the physical and mental condition of the patient."
4. Medical progress is based on research which ultimately must rest in part on experimentation involving human subjects.
5. In medical research on human subjects, considerations related to the well-being of the human subject should take precedence over the interests of science and society.
6. The primary purpose of medical research involving human subjects is to improve prophylactic, diagnostic and therapeutic procedures and the understanding of the etiology and pathogenesis of disease. Even the best proven prophylactic, diagnostic, and therapeutic methods must continuously be challenged through research for their effectiveness, efficiency, accessibility and quality.
7. In current medical practice and in medical research, most prophylactic, diagnostic and therapeutic procedures involve risks and burdens.

8. Medical research is subject to ethical standards that promote respect for all human beings and protect their health and rights. Some research populations are vulnerable and need special protection. The particular needs of the economically and medically disadvantaged must be recognized. Special attention is also required for those who cannot give or refuse consent for themselves, for those who may be subject to giving consent under duress, for those who will not benefit personally from the research and for those for whom the research is combined with care.
9. Research Investigators should be aware of the ethical, legal and regulatory requirements for research on human subjects in their own countries as well as applicable international requirements. No national ethical, legal or regulatory requirement should be allowed to reduce or eliminate any of the protections for human subjects set forth in this Declaration.

B. BASIC PRINCIPLES FOR ALL MEDICAL RESEARCH

10. It is the duty of the physician in medical research to protect the life, health, privacy, and dignity of the human subject.
11. Medical research involving human subjects must conform to generally accepted scientific principles, be based on a thorough knowledge of the scientific literature, other relevant sources of information, and on adequate laboratory and, where appropriate, animal experimentation.
12. Appropriate caution must be exercised in the conduct of research which may affect the environment, and the welfare of animals used for research must be respected.
13. The design and performance of each experimental procedure involving human subjects should be clearly formulated in an experimental protocol. This protocol should be submitted for consideration, comment, guidance, and where appropriate, approval to a specially appointed ethical review committee, which must be independent of the investigator, the sponsor or any other kind of undue influence. This independent committee should be in conformity with the laws and regulations of the country in which the research experiment is performed. The committee has the right to monitor ongoing trials. The researcher has the obligation to provide monitoring information to the committee, especially any serious adverse events. The researcher should also submit to the committee, for review, information regarding funding, sponsors, institutional affiliations, other potential conflicts of interest and incentives for subjects.
14. The research protocol should always contain a statement of the ethical considerations involved and should indicate that there is compliance with the principles enunciated in this Declaration.
15. Medical research involving human subjects should be conducted only by scientifically qualified persons and under the supervision of a clinically competent medical person. The responsibility for the human subject must always rest with a medically qualified person and never rest on the subject of the research, even though the subject has given consent.

16. Every medical research project involving human subjects should be preceded by careful assessment of predictable risks and burdens in comparison with foreseeable benefits to the subject or to others. This does not preclude the participation of healthy volunteers in medical research. The design of all studies should be publicly available.
17. Physicians should abstain from engaging in research projects involving human subjects unless they are confident that the risks involved have been adequately assessed and can be satisfactorily managed. Physicians should cease any investigation if the risks are found to outweigh the potential benefits or if there is conclusive proof of positive and beneficial results.
18. Medical research involving human subjects should only be conducted if the importance of the objective outweighs the inherent risks and burdens to the subject. This is especially important when the human subjects are healthy volunteers.
19. Medical research is only justified if there is a reasonable likelihood that the populations in which the research is carried out stand to benefit from the results of the research.
20. The subjects must be volunteers and informed participants in the research project.
21. The right of research subjects to safeguard their integrity must always be respected. Every precaution should be taken to respect the privacy of the subject, the confidentiality of the patient's information and to minimize the impact of the study on the subject's physical and mental integrity and on the personality of the subject.
22. In any research on human beings, each potential subject must be adequately informed of the aims, methods, sources of funding, any possible conflicts of interest, institutional affiliations of the researcher, the anticipated benefits and potential risks of the study and the discomfort it may entail. The subject should be informed of the right to abstain from participation in the study or to withdraw consent to participate at any time without reprisal. After ensuring that the subject has understood the information, the physician should then obtain the subject's freely-given informed consent, preferably in writing. If the consent cannot be obtained in writing, the non-written consent must be formally documented and witnessed.
23. When obtaining informed consent for the research project the physician should be particularly cautious if the subject is in a dependent relationship with the physician or may consent under duress. In that case the informed consent should be obtained by a well-informed physician who is not engaged in the investigation and who is completely independent of this relationship.
24. For a research subject who is legally incompetent, physically or mentally incapable of giving consent or is a legally incompetent minor, the investigator must obtain informed consent from the legally authorized representative in accordance with applicable law. These groups should not be included in research unless the research is necessary to promote the health of the population represented and this research cannot instead be performed on legally competent persons.

25. When a subject deemed legally incompetent, such as a minor child, is able to give assent to decisions about participation in research, the investigator must obtain that assent in addition to the consent of the legally authorized representative.

26. Research on individuals from whom it is not possible to obtain consent, including proxy or advance consent, should be done only if the physical/mental condition that prevents obtaining informed consent is a necessary characteristic of the research population. The specific reasons for involving research subjects with a condition that renders them unable to give informed consent should be stated in the experimental protocol for consideration and approval of the review committee. The protocol should state that consent to remain in the research should be obtained as soon as possible from the individual or a legally authorized surrogate.

27. Both authors and publishers have ethical obligations. In publication of the results of research, the investigators are obliged to preserve the accuracy of the results. Negative as well as positive results should be published or otherwise publicly available. Sources of funding, institutional affiliations and any possible conflicts of interest should be declared in the publication. Reports of experimentation not in accordance with the principles laid down in this Declaration should not be accepted for publication.

C. ADDITIONAL PRINCIPLES FOR MEDICAL RESEARCH COMBINED WITH MEDICAL CARE

28. The physician may combine medical research with medical care, only to the extent that the research is justified by its potential prophylactic, diagnostic or therapeutic value. When medical research is combined with medical care, additional standards apply to protect the patients who are research subjects.

29. The benefits, risks, burdens and effectiveness of a new method should be tested against those of the best current prophylactic, diagnostic, and therapeutic methods. This does not exclude the use of placebo, or no treatment, in studies where no proven prophylactic, diagnostic or therapeutic method exists.

30. At the conclusion of the study, every patient entered into the study should be assured of access to the best proven prophylactic, diagnostic and therapeutic methods identified by the study.

31. The physician should fully inform the patient which aspects of the care are related to the research. The refusal of a patient to participate in a study must never interfere with the patient-physician relationship.

32. In the treatment of a patient, where proven prophylactic, diagnostic and therapeutic methods do not exist or have been ineffective, the physician, with informed consent from the patient, must be free to use unproven or new prophylactic, diagnostic and therapeutic measures, if in the physician's judgement it offers hope of saving life, re-establishing health or alleviating suffering. Where possible, these measures should be made the object of research, designed to evaluate their safety and efficacy. In all cases, new information should be recorded and, where appropriate, published. The other relevant guidelines of this Declaration should be followed.

23.8 LABELLING

Exemple d'étiquette du "medication box"

Chaque capsule contient 3,5 mg d'extrait bactérien ou de placebo

Prendre une capsule par jour avec le repas.
La capsule peut être diluée dans un liquide (eau/jus/lait).
Ne pas oublier de rapporter l'emballage à la prochaine visite.

Utilisation sous stricte surveillance médicale (Art.5123 CSP)
Médicament pour essai clinique.

A conserver entre 15 – 25°C
Tenir hors de portée des enfants.

ETUDE CLINIQUE BV-2002 / 1
IMOCUR 3,5 mg / PLACEBO
30 capsules
MOIS : X TRAITEMENT N°: «PAT_1».
LOT N° : XXXXX EXP.: XX / XXXX
Initiales du patient :__________
Date de dispensation :___/___/___/
OM PHARMA MEYRIN GENEVE SUISSE

ETUDE CLINIQUE BV-2002 / 1
IMOCUR 3,5 mg / PLACEBO
30 capsules
MOIS RESERVE TRAITEMENT N°: «PAT_1»
LOT N° : XXXXX EXP.: XX / XXXX
Initiales du patient :__________
Date de dispensation :___/___/___/
OM PHARMA MEYRIN GENEVE SUISSE

Exemple d'étiquette du "patient kit"

ETUDE CLINIQUE BV-2002 / 1
Traitement N° «PAT_1»

IMOCUR / PLACEBO
LOT N° XXXXX EXP. : XX / XXXX
Chaque capsule contient 3,5 mg d'extrait bactérien ou de placebo
10 boites mensuelles de 30 capsules = 300 capsules
(Mois 1 à 9 + 1 réserve)
Utilisation sous stricte surveillance médicale
(Art. 5123 CSP) : Médicament pour essai clinique.

A conserver entre 15 - 25°C
Tenir hors de la portée des enfants
OM PHARMA MEYRIN / GENEVE SUISSE

Exemple d'étiquette du "centre box"

ETUDE CLINIQUE BV – 2002 / 1

IMOCUR 3,5 mg / PLACEBO

CENTRE : .....

TRAITEMENTS N° : XX à N° : XX

LOT N° XXXXX EXP.: XX / XXXX

XX TRAITEMENTS = XXXX CAPSULES

UTILISATION SOUS STRICTE SURVEILLANCE MEDICALE (Art.5123 CSP)
MEDICAMENT POUR ESSAI CLINIQUE
A CONSERVER ENTRE :15 °C – 25 °C
A TENIR HORS DE PORTEE DES ENFANTS

OM PHARMA MEYRIN GENEVE SUISSE

Exemple d'inscriptions sur le "pharmaceutical packing"

BV – 2002 / 1

Imocur 3,5 mg / Placebo

Traitement N° : « PAT_1 »

Lot N° XXXXX Exp. : XX/XXXX

OM PHARMA MEYRIN GENEVE SUISSE

23.9 OBLIGATIONS OF SPONSOR AND INVESTIGATOR

SPONSOR'S OBLIGATIONS

The sponsor has the responsibility:

- to notify the study to the appropriate regulatory authorities and to submit any required applications after obtaining the favorable opinion of the Ethics Committee.
- to provide the investigator with all the materiel needed for the study according to the protocol.
- to inform the investigator of any new important information on the study drug becoming available during the study duration.
- to announce the serious adverse events/drug reactions (SAEs) to the regulatory authorities according to the national legislation.
- to reserve the right to terminate the study at any time and to ask the investigator to exclude a patient in case of a major protocol violation.

Investigator's obligations

The investigator agrees:

- to adhere to the principles laid down in the Declaration of Helsinki and its amendments as well as to the guidelines of GCP.
- to submit the trial documentation (protocol, informed consent, Investigator's Brochure etc.) to the responsible Ethics Committee before the start of the study, to provide a copy of their opinion to the sponsor as well as a statement confirming that the Ethics Committee is organized and operates according to GCP and applicable laws and regulations.
- to provide a short Curriculum vitae (CV) with clinical research experience before starting the study.
- to conduct the study in accordance with the relevant, current protocol. He will only make changes in a protocol after notifying the sponsor and Ethics Committee, except when necessary to protect the safety, the rights or welfare of subjects.
- to personally conduct or supervise the study.
- to ensure that all co-investigators, research nurses and employees assisting in the study are informed about their obligations.
- to ensure the patient recruitment. In case of difficulties, to discuss as soon as possible with the study monitor.
- to inform any child's parent/legal guardian that the drug is being used for investigation purposes and to obtain their dated, signed informed consent.
- to complete and sign the CRFs for each patient included in the trial in accordance with the raw data.
- to keep an up-to-date list of the selected patients, screened or enrolled (patient log).
- to ensure the storage of the trial drug in a place with limited access as specified by the sponsor.
- to complete regularly the drug accountability form.
- to report serious adverse events/adverse drug reactions, whether considered to be related to the study drug/study procedure or not, according to the procedures described in the protocol, to the sponsor immediately and to the Ethics Committee and to the relevant regulatory authorities according to the national/local requirements.

- to maintain the subject's confidentiality.
- to make patient records available to the study monitor and/or person mandated for audit or inspection.
- to archive the patient identification list (patient log) and the signed dated informed consent forms during at least 15 years.
- to archive the study records as long as the national legislation requires or as specified by the sponsor.
