# Supplementary material for: Adjuvant treatment with the bacterial lysate (OM-85) improves management of atopic dermatitis: A randomized study
Source: PLoS One. 2017 Mar 23;12(3):e0161555. doi: 10.1371/journal.pone.0161555 (PMC5363804; doi:10.1371/journal.pone.0161555)
Supplement: S1 Table — (DOCX) [file pone.0161555.s003.docx]

**S1 Table**. **Enrolment, patients baseline and follow-up characteristics, included atopic dermatitis (AD) clinical evaluation, by treatment group.**

|  | **OM-85**  **(N=88)** | | **PLACEBO**  **(N=82)** | |
| --- | --- | --- | --- | --- |
|  | **N** | **(%)** | **N** | **(%)** |
| ***Enrolment characteristics*** |  |  |  |  |
| **Centre** |  |  |  |  |
| Hospitals | 63 | (71.6) | 62 | (75.6) |
| Private practitioners | 25 | (28.4) | 20 | (24.4) |
| **Month of enrolment** |  |  |  |  |
| January-March | 19 | (21.6) | 16 | (19.5) |
| April-June | 22 | (25.0) | 26 | (31.7) |
| July-September | 23 | (26.1) | 17 | (20.7) |
| October-December | 24 | (27.3) | 23 | (28.1) |
| ***Patient characteristics*** |  |  |  |  |
| **Sex** |  |  |  |  |
| Male | 51 | (58.0) | 58 | (70.7) |
| Female | 37 | (42.0) | 24 | (29.3) |
| **Age (years)^a^** |  |  |  |  |
| ≤3 | 59 | (67.0) | 58 | (70.7) |
| >3 | 29 | (33.0) | 24 | (29.3) |
| N Mean, Median (q1-q3) | 88 | 2.4, 2.0 (0.9-3.6) | 82 | 2.5, 1.6 (1.0-3.8) |
| **Height (cm)** |  |  |  |  |
| N Mean, Median (q1-q3) | 86 | 86.2, 86.0 (73.0-98.0) | 80 | 87.0, 85.0 (73.0-102.0) |
| **Weight (kg)** |  |  |  |  |
| N Mean, Median (q1-q3) | 87 | 12.4, 12.0 (8.7-14.3) | 81 | 12.4, 11.0 (9.0-15.0) |
| **Body Surface Area (m^2^)^b^** |  |  |  |  |
| N Mean, Median (q1-q3) | 86 | 0.5, 0.5 (0.4-0.6) | 80 | 0.5, 0.5 (0.4-0.7) |
| **Family history of atopy in parents** |  |  |  |  |
| No | 29 | (33.0) | 26 | (31.7) |
| Yes | 59 | (67.0) | 56 | (68.3) |
| **Family history of AD in parents** |  |  |  |  |
| No | 37 | (42.0) | 35 | (42.7) |
| Yes | 51 | (58.0) | 47 | (57.3) |

*Continues…..*

*…..Continued*

|  | **OM-85**  **(N=88)** | | **PLACEBO**  **(N=82)** | | |
| --- | --- | --- | --- | --- | --- |
|  | **N** | **(%)** | **N** | **(%)** | |
| ***AD: clinical evaluation*** |  |  |  | |  |
| **Months since first symptoms** |  |  |  | |  |
| ≤18 | 42 | (49.4) | 42 | | (51.2) |
| >18 | 43 | (50.6) | 40 | | (48.8) |
| **Number of flares during the last year** |  |  |  | |  |
| ≤8 | 39 | (53.4) | 32 | | (47.8) |
| >8 | 34 | (46.6) | 35 | | (52.2) |
| N Mean, Median (q1-q3) | 73 | 12.7, 7.0 (4.0-12.0) | 67 | | 10.8, 10.0 (4.0-12.0) |
| **Intensity (parents)** |  |  |  | |  |
| No symptoms | 0 | (0.0) | 0 | | (0.0) |
| Low | 3 | (3.4) | 7 | | (8.5) |
| Moderate | 39 | (44.3) | 38 | | (46.3) |
| Important | 39 | (44.3) | 28 | | (34.2) |
| Very important | 7 | (8.0) | 9 | | (11.0) |
| **Intensity (investigator)** |  |  |  | |  |
| No symptoms | 0 | (0.0) | 0 | | (0.0) |
| Low | 0 | (0.0) | 1 | | (1.2) |
| Moderate | 64 | (72.7) | 56 | | (68.3) |
| Important | 22 | (25.0) | 20 | | (24.4) |
| Very important | 2 | (2.3) | 5 | | (6.1) |
| **SCORAD** |  |  |  | |  |
| N Mean, Median (q1-q3) | 88 | 41.1, 39.9 (32.8-47.6) | 82 | | 43.5, 42.2 (33.7-51.0) |
| <40 (Block A) | 44 | 32.6, 32.8 (29.5-36.9) | 35 | | 32.0, 31.5 (28.4-35.5) |
| ≥40 (Block B) | 44 | 49.7, 47.6 (43.2-55.2) | 47 | | 52.2, 49.0 (44.0-59.3) |
| ***Follow-up description*** |  |  |  | |  |
| **Total Follow-up time (days)**  N Mean, Median (q1-q3) | 88 | 261.0, 273 (266-279) | 82 | | 247.3, 273 (261-279) |
| **Number of visits per patient** |  |  |  | |  |
| 1 (V2: 1 month) | 88 | (100) | 82 | | (100) |
| 2 (V3: 3 months) | 85 | (96.6) | 76 | | (92.7) |
| 3 (V4: 6 months) | 82 | (93.2) | 74 | | (91.5) |
| 4 (V5: 9 months) | 80 | (90.9) | 70 | | (85.4) |

q1=first quartile (25° percentile); q3=third quartile (75° percentile).

**^a^** Infants younger than one year old were 52 (30% of the total 170 children), 29 (33%) in the OM-85 group and 23 (28%) in the placebo group.

**^b^** Body Surface Area [m^2^] was calculated according to Mosteller formula ([Height (cm) x Weight (kg)]/3600)½.
