# Supplementary material for: Adjuvant treatment with the bacterial lysate (OM-85) improves management of atopic dermatitis: A randomized study
Source: PLoS One. 2017 Mar 23;12(3):e0161555. doi: 10.1371/journal.pone.0161555 (PMC5363804; doi:10.1371/journal.pone.0161555)
Supplement: S2 Table — (Q test p value for heterogeneity between HR estimates was showed for subgroups). (DOCX) [file pone.0161555.s004.docx]

**S2 Table. Hazard ratios (HR) estimates for treatment effect, obtained applying the multiple-events model (and their 95% confidence intervals (CI)), in subgroups of patients monitored up to the last follow-up treatment visit (9 months after randomization). (Q test p value for heterogeneity between HR estimates was showed for subgroups).**

|  | **HR^a^ (95% CI)** | **HR^b^ (95% CI)** |
| --- | --- | --- |
| **Subgroups** |  |  |
| **Sex** |  |  |
| Male | 0.78 (0.63-0.97) | 0.77 (0.62-0.94) |
| Female | 0.93 (0.67-1.27) | 0.93 (0.68-1.28) |
| *Q test for heterogeneity (p value)* | *0.37* | *0.30* |
| **Age (years)** |  |  |
| ≤3 | 0.78 (0.63-0.97) | 0.79 (0.64-0.97) |
| >3 | 0.85 (0.62-1.16) | 0.75 (0.53-1.06) |
| *Q test for heterogeneity (p value)* | *0.66* | *0.75* |
| **Family history of atopy in parents** |  |  |
| No | 0.77 (0.54-1.11) | 0.75 (0.53-1.08) |
| Yes | 0.79 (0.64-0.96) | 0.75 (0.61-0.92) |
| *Q test for heterogeneity (p value)* | *0.90* | *0.99* |
| **SCORAD at entry** |  |  |
| < 40 | 0.80 (0.61-1.06) | 0.70 (0.52-0.95) |
| > 40 | 0.85 (0.68-1.07) | 0.85 (0.68-1.07) |
| *Q test for heterogeneity (p value)* | *0.88* | *0.55* |

**^a^** Crude analysis.

**^b^** Adjusted when appropriate, for age, sex, history of atopy in parents, and total corticosteroids use.
