# Supplementary material for: Adjuvant treatment with the bacterial lysate (OM-85) improves management of atopic dermatitis: A randomized study
Source: PLoS One. 2017 Mar 23;12(3):e0161555. doi: 10.1371/journal.pone.0161555 (PMC5363804; doi:10.1371/journal.pone.0161555)
Supplement: S3 Table — (DOCX) [file pone.0161555.s005.docx]

**S3 Table. Distribution of adverse events (and concomitant diseases) as percentages of events and list of adverse events with probable/possible relationship with OM-85 or placebo.**

| **Distribution of adverse events (and concomitant diseases) as percentages of events** | | | | | | **OM-85** | **Placebo** | **Total** |
| --- | --- | --- | --- | --- | --- | --- | --- | --- |
| Respiratory, thoracic and mediastinal disorders | | | | | | 55.646% | 54.013% | 54.858% |
| Gastrointestinal disorders | | | | | | 14.617% | 15.687% | 15.133% |
| General disorders and administration site conditions | | | | | | 7.641% | 8.913% | 8.255% |
| Ear and labyrinth disorders | | | | | | 7.475% | 8.200% | 7.825% |
| Infections and infestations | | | | | | 5.980% | 3.030% | 4.557% |
| Skin and subcutaneous tissue disorders | | | | | | 2.824% | 3.030% | 2.923% |
| Eye disorders | | | | | | 2.159% | 1.961% | 2.064% |
| Injury and poisoning | | | | | | 1.163% | 2.496% | 1.806% |
| Musculoskeletal, connective tissue and bone disorders | | | | | | 0.664% | 0.357% | 0.516% |
| Psychiatric disorders | | | | | | 0.664% | 0.178% | 0.430% |
| Immune system disorders | | | | | | 0.332% | 0.357% | 0.344% |
| Reproductive system and breast disorders | | | | | | 0.000% | 0.713% | 0.344% |
| Renal and urinary disorders | | | | | | 0.332% | 0.178% | 0.258% |
| Surgical and medical procedures | | | | | | 0.166% | 0.357% | 0.258% |
| Metabolism and nutrition disorders | | | | | | 0.166% | 0.178% | 0.172% |
| Nervous system disorders | | | | | | 0.000% | 0.086% | 0.086% |
| Vascular disorders | | | | | | 0.166% | 0.000% | 0.086% |
| Congenital and familial/genetic disorders | | | | | | 0.000% | 0.178% | 0.086% |
| Cardiac disorders | | | | | | --- | --- | --- |
| Endocrine disorders | | | | | | --- | --- | --- |
| Hepato-biliary disorders | | | | | | --- | --- | --- |
| Investigations | | | | | | --- | --- | --- |
| Neoplasms benign and malignant (including cysts and polyps) | | | | | | --- | --- | --- |
| Pregnancy, puerperium and perinatal conditions | | | | | | --- | --- | --- |
|  | | | | | | 100% | 100 % | 100 % |
| **Adverse events with probable/possible relationship with OM-85 or placebo** | | | | | | | | |
| **Pat.#** | **Group** | **Description** | **Relationship** | **Onset [day]**  **from start** | **Severity** | **Action taken** | **Outcome** | **Sequelae** |
| 133 | OM-85 | Diarrhoea  (ICD 787.91) | Possible | 34 | Moderate | Other therapy | Resolved | None |
| 182 | Placebo | Diarrhoea  (ICD 787.91) | Possible | 56 | Moderate | Same | Resolved | None |
| 240 | OM-85 | Agitation  (ICD: 308.2) | Possible | 292 | Mild | Reduced | Resolved | None |
| 248 | OM-85 | Cough  (ICD 786.2) | Possible | 63 | Severe | Other therapy | Unknown | None |
| 297 | Placebo | Diarrhoea  (ICD 787.91) | Possible | 231 | moderate | Same | Resolved | None |
